# **A multi-molecular biomarker assessment of thermal preconditioning in two scleractinian coral species**

**DOI:** 10.1038/s41598-025-18617-3

**Published:** 2025-10-06

**Authors:** Yohan Didier Louis, Enrico Montalbetti, Valerio Isa, Davide Maggioni, Ludovico Pravettoni, Rebecca Pollutri, Jacopo Gobbato, Simone Montano, Ivan Orlandi, Marina Vai, Silvia Lavorano, Paolo Galli, Davide Seveso

**Affiliations:** 1 Department of Earth and Environmental Science, University of Milan-Bicocca, Piazza della Scienza 1, Milan, 20126 Italy; 2 MaRHE Center (Marine Research and High Education Center), Magoodhoo Island, Faafu Atoll, Republic of Maldives; 3 Department of Biotechnology and Biosciences, University of Milan-Bicocca, Piazza della Scienza 2, Milan, 20126 Italy; 4 NBFC (National Biodiversity Future Center), Palermo, 90133 Italy; 5 Costa Edutainment SpA - Acquario di Genova, Area Porto Antico, Ponte Spinola, Genoa, 16128 Italy

**Keywords:** Thermal preconditioning, Coral bleaching, Heat shock proteins, Hsp70, Oxidative damage, *Pocillopora damicornis*, *Stylophora pistillata*, Ocean sciences, Marine biology, Molecular biology

## Abstract

Coral reefs face escalating threats from rising sea temperatures, triggering widespread mass bleaching and mortality events. Thermal preconditioning, a process in which corals exposed to sublethal thermal stress become more tolerant to subsequent thermal stress, is increasingly recognised as a promising strategy for restoration purposes. However, its underlying mechanisms remain unclear. Furthermore, species-specific responses to thermal preconditioning must be understood to ensure its broad applicability. In this study, we conducted a comprehensive multi-molecular biomarker analysis to investigate the physiological and molecular effects of thermal preconditioning on two coral species, *Pocillopora damicornis* and *Stylophora pistillata*, by exposing them to a sublethal thermal stress of + 3 °C above ambient temperature, followed by an acute stress of 32 °C (+ 8 °C). In both species, preconditioned corals showed delayed bleaching, maintaining chlorophyll *a* and *c2* concentrations, along with Symbiodiniaceae density, for longer periods compared to non-preconditioned corals. At the molecular level, this increased thermal tolerance could be linked to the higher activity of antioxidant enzymes measured, suggesting enhanced protection against oxidative stress and consequently lower levels of lipid peroxidation (LPO) damage. Preconditioned corals also exhibited a slower downregulation of Hsp70 or sustained Hsp70 expression above basal levels during acute heat stress, possibly reflecting prolonged cellular homeostasis. Additionally, they demonstrated delayed or reduced *hsp70* gene expression, indicating a less immediate response to thermal stress. Furthermore, the acquired thermal tolerance lasted longer in *P. damicornis*, highlighting a species-specific response to thermal preconditioning. Our study provides crucial molecular insights into the complex mechanisms of thermal preconditioning, which will be essential for developing more effective and species-targeted preconditioning protocols for practical application in coral reef restoration efforts.

**Supplementary Information:**

The online version contains supplementary material available at 10.1038/s41598-025-18617-3.

## Introduction

Coral reefs, recognised as some of the most functional and biodiverse ecosystems on the planet, are increasingly at risk due to climate change related rising sea temperatures, which have triggered widespread bleaching events and substantial coral mortality^1^. It is projected that by mid-century, 70–90% of global coral populations could be lost^2^. In response, numerous interventions have been implemented, including carbon dioxide emission reduction treaties, the establishment of marine protected areas, and habitat restoration efforts^3^. One such intervention is coral farming, a widely adopted restoration technique, which involves cultivating coral fragments in controlled environments before transplanting them onto degraded reefs, aiding in reef recovery^4,5^. However, as climate change apace rapidly, mass bleaching events, which have already increased in their frequency and intensity in the last decades^6,7^, are anticipated to occur annually across most coral habitats by 2030–2060^8–11^. This presents a significant challenge, as transplanted corals may not survive the increasingly frequent temperature extremes predicted^12^. To overcome this, innovative strategies such as selective breeding, assisted gene flow, and coral microbiome manipulation are being explored to enhance coral resistance and resilience^13^.

Thermal preconditioning involves subjecting corals to non-lethal heat stress to increase their thermal tolerance^14–17^. Field evidence suggests that corals exposed to previous thermal stress, bleaching events or fluctuating temperature regimes exhibit greater tolerance to subsequent heat stress, potentially indicating a form of stress memory^18,19^. Experimental studies further support the efficacy of thermal preconditioning, demonstrating significant improvements in coral tolerance to bleaching conditions^20^. Despite these encouraging findings, the underlying mechanisms and optimal thermal preconditioning protocols remain poorly understood. Many experimental studies on preconditioning have focused on observable outcomes, such as reduced bleaching and improved physiological performance^20–24^, as well as the determination of optimal temperature regimes^25^. Further research has aimed to elucidate the mechanisms underlying this acquired thermal tolerance across various biological levels. For instance, studies on *Acropora millepora*^26^, *Acropora nana*^27^, and *Montipora capitata*^28^ have examined changes in gene expression as potential contributors to enhanced thermal tolerance. The involvement of bacterial and algal symbionts in this process has also been explored, particularly in *A. millepora*^29^. Other studies have focused on the coral antioxidant responses triggered by thermal preconditioning, such as the increased in glutathione reductase (GR) that accompanied increased in tolerance in *Pocillopora acuta* following thermal preconditioning^30^. Similarly, Huang et al. (2024) reported increased antioxidant enzyme activity (catalase and superoxide dismutase), along with enhanced ammonium assimilation (glutamine synthetase) and elevated apoptosis markers (lipid peroxide and caspase-3). Additionally, Majerová et al. (2021) demonstrated that in preconditioned *P. acuta*, the ratio of pro-survival genes (pa-Bcl-2 and pa-BI-1) to pro-death genes (pa-BAK and pa-BAX) increased, correlating with significantly reduced bleaching. Other aspects such as the nutrient metabolism in preconditioned corals were also investigated^31^, further contributing to our understanding of the complex mechanisms involved in coral preconditioning.

However, even with over a decade of research, the preconditioning process remains insufficiently understood^24,32^. This is largely due to (1) the complexity of biological pathways involved in coral bleaching susceptibility and thermal tolerance^33^, and (2) the reduced number of coral species investigated make it challenging to generalise findings across broader taxonomic groups, as not all coral species may benefit equally from thermal preconditioning. To reach a consensus on the efficacy and mechanisms involved in thermal preconditioning, further research is needed across a diverse range of coral species and should target different cellular and molecular pathways involved in the acquired thermotolerance.

In this study, we focused on *Stylophora pistillata* and *Pocillopora damicornis*, two widely distributed tropical coral species that are among the three most used in coral restoration^4^. Despite their widespread distribution and use, these species have been relatively understudied in terms of thermal preconditioning. We conducted a comprehensive multi-molecular biomarker analysis to better understand whether and how thermal preconditioning may enhance bleaching tolerance in these two coral species. We exposed both species to a sublethal thermal stress of + 3 °C above ambient temperature, followed by an acute stress of 32 °C (+ 8 °C). By measuring gene, protein, and enzyme levels, we aimed to provide a thorough understanding of the molecular mechanisms underlying thermal tolerance. This multi-biomarker approach could be more advantageous because it allows for the simultaneous assessment of multiple cellular and molecular responses, including the interactions between pathways that contribute to thermal tolerance. In this context, many studies have highlighted the critical role of heat shock proteins (Hsps) and antioxidant enzymes in enhancing coral tolerance to heat stress and bleaching^15,34^. Heat shock proteins act as molecular chaperones to maintain protein structure and functionality under stress conditions, while antioxidant enzymes, such as superoxide dismutase (SOD), catalase (CAT), and glutathione reductase, mitigate the harmful effects of reactive oxygen species (ROS), over-produced during heat stress events^35,36^. By investigating these critical molecular biomarkers simultaneously, together with coral bleaching proxies such as the photosynthetic symbiont (Symbiodiniaceae) density and chlorophyll concentration, our study could offer a more holistic understanding of thermal preconditioning.

## Results

### Thermal preconditioning delays bleaching in both *P. damicornis* and *S. pistillata*

**Fig. 1:**
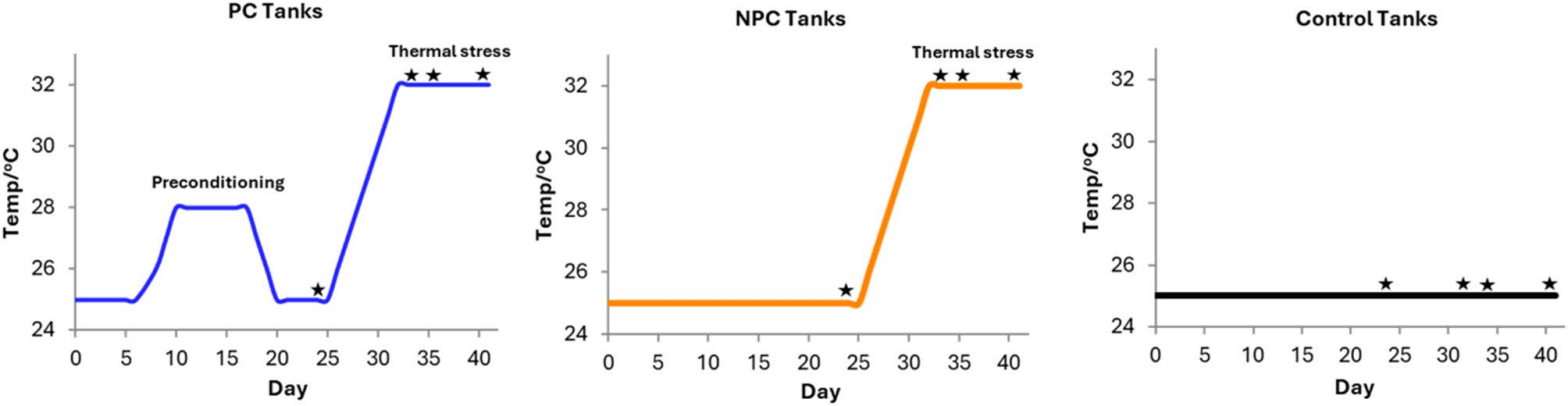
Temperature profiles and sampling points used in the experiment.

Coral nubbins were exposed to thermal preconditioning: a sublethal thermal stress of + 3 °C above ambient temperature (25 °C), followed by an acute stress of + 8 °C (32 °C). A non-preconditioned group was exposed directly to the acute stress, while the control group was maintained at ambient temperature throughout the experiment. Biomarkers were measured at four time points: before acute stress, Day 1, Day 3, and Day 10 after reaching the acute thermal stress of 32 °C (Fig. 1).

**Fig. 2:**
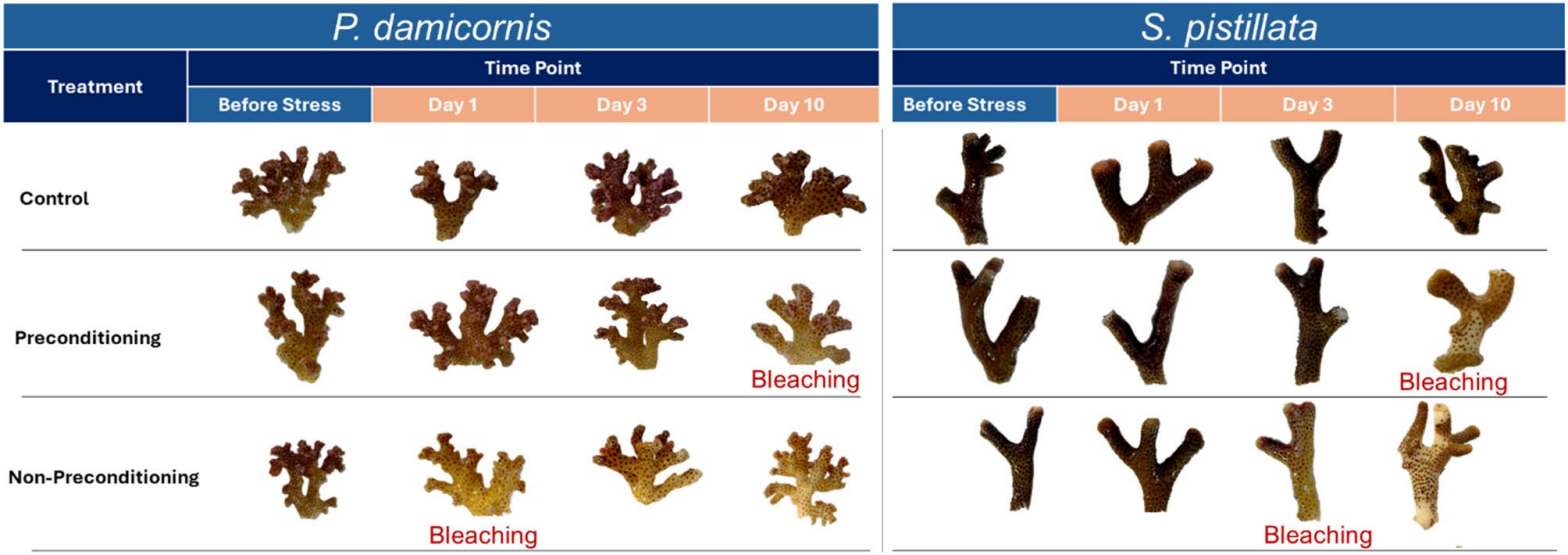
Rapid visual bleaching assessment of *P. damicornis* and *S. pistillata* nubbins under three different treatments at specific time intervals (before stress, and after 1, 3, 10 days after acute thermal stress), with a representative sample shown for each time point. Bleaching was considered to have occurred at a given time point when at least 7 out of 8 coral nubbins within a treatment group exhibited visible signs of bleaching. This rapid visual assessment was then validated through quantitative bleaching measurements.

Rapid visual assessment of coral colonies revealed that non-preconditioned (NPC) corals of both species bleached earlier than preconditioned (PC) nubbins (Fig. 2). For *P. damicornis*, non-preconditioned corals showed sign of bleaching as early as Day 1 following thermal stress, while preconditioned corals showed signs of bleaching only by Day 10. Similarly, non-preconditioned *S. pistillata* nubbins exhibited bleaching on Day 3, whereas preconditioned ones did not bleach until Day 10 (Fig. 2). Necrosis was also observed on Day 10 for both preconditioned and non-preconditioned *S. pistillata*.

**Fig. 3:**
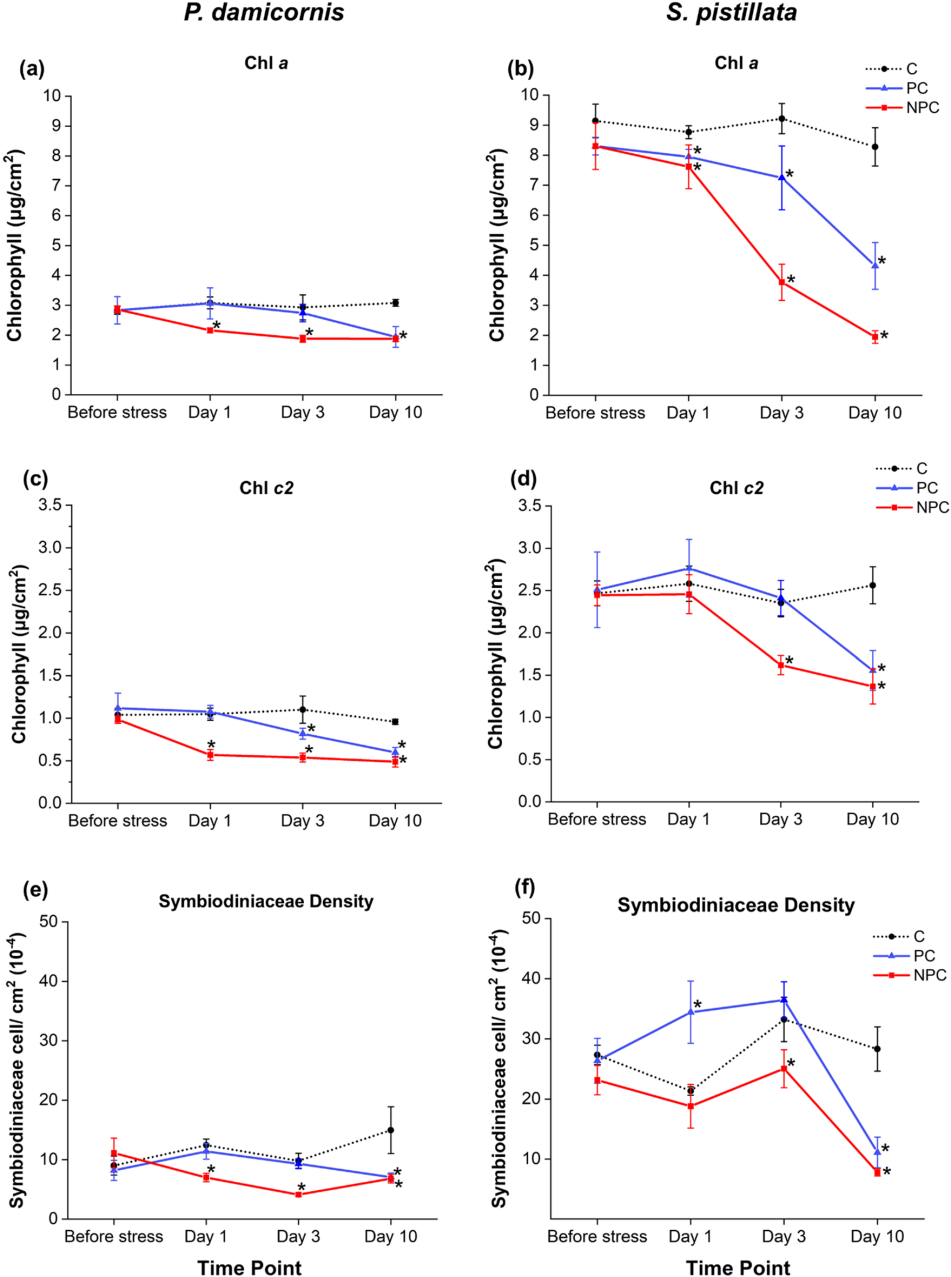
Concentrations of chlorophyll *a* (Chl *a*), chlorophyll *c2* (Chl *c2*), and Symbiodiniaceae density in *P. damicornis* **(a**,** c**,** e)** and *S. pistillata* **(b**,** d**,**f)** under three different treatments (C, PC and NPC) at specific time intervals (Before stress, Day 1, Day 3, Day 10). Asterisks indicate statistically significant differences (*p* < 0.05) from the control group (C) at the corresponding time points, as determined by the two-factor univariate PERMANOVA followed by pairwise comparisons. Error bars represent the standard error of the mean. Full PERMANOVA pairwise comparisons are provided in Table S2 and S4 (Supplementary Information).

To quantify the observed extend of bleaching, the concentrations of chlorophyll *a* (Chl *a*), chlorophyll *c2* (Chl *c2*), and Symbiodiniaceae density were measured. In *P. damicornis*, both Chl *a* and *c2* concentration and Symbiodiniaceae density showed significant differences between the different treatments (PC, NPC and Control; Supplementary Information, Table S1). Moreover, the concentration of Chl *c2* significantly changed between the different sampling time points (Before stress, Day 1, 3 and 10) and as a result of the interaction of both the investigated factors (Treatment x Time) (Table S1). The latter also significantly affected the Symbiodiniaceae density (Table S1). Specifically, the concentrations of Chl *a*, Chl *c2*, and Symbiodiniaceae density remained stable in the control group throughout the experiment (Fig. 3a,c,e). In non-preconditioned corals, however, a significant decrease in Chl *a*, Chl *c2*, and Symbiodiniaceae density was observed already on Day 1 post-acute heat stress compared to controls, with this decline persisting until Day 10 (Table S2). By Day 10, Symbiodiniaceae density had increased relative to Day 3 but remained lower than both the control group and before stress.

Conversely, preconditioned corals did not show a significant decrease in Chl *a* or Symbiodiniaceae density until Day 3 compared to controls (Table S2). However, Chl *c2* concentration was significantly lower than in the control group on Day 3, though it remained higher than in non-preconditioned corals (Table S2). By Day 10, Chl *a*, Chl *c2*, and Symbiodiniaceae density levels had decreased relative to controls and were similar to those observed in non-preconditioned corals.

In *S. pistillata*, a different trend was observed compared to *P. damicornis* (Fig. 3). Overall, in *S. pistillata* a significant effect of treatments, sampling time points and the interaction of both factors was recorded for Chl *a* and *c2* concentrations, and Symbiodiniaceae density (Table S3). In particular, in non-preconditioned *S. pistillata* colonies, neither the Chl *c2* concentration nor Symbiodiniaceae density showed a significant decrease on Day 1 compared to controls. Chl *a* was significantly lower than in the control corals but showed no significant difference compared to the preconditioned corals (Table S4). However, by Day 3, all three parameters were significantly lower than controls. The concentrations of Chl *a*, Chl *c2* and, Symbiodiniaceae density continued to decline through Day 10 (Fig. 3b, d, f; Table S4).

In preconditioned corals, no significant decrease in Chl *c2* nor in Symbiodiniaceae density was observed on Days 1 and 3 compared to controls, only a decrease in Chl *a* was observed on Day 3 (Table S4). A significant reduction in all three parameters was observed after 10 days. However, these levels remained higher compared to non-preconditioned corals. In the control group, concentrations of Chl *a* and Chl *c2* remained relatively stable throughout the experiment (Table S4).

### Preconditioned corals maintained higher antioxidant enzyme activities

**Fig. 4:**
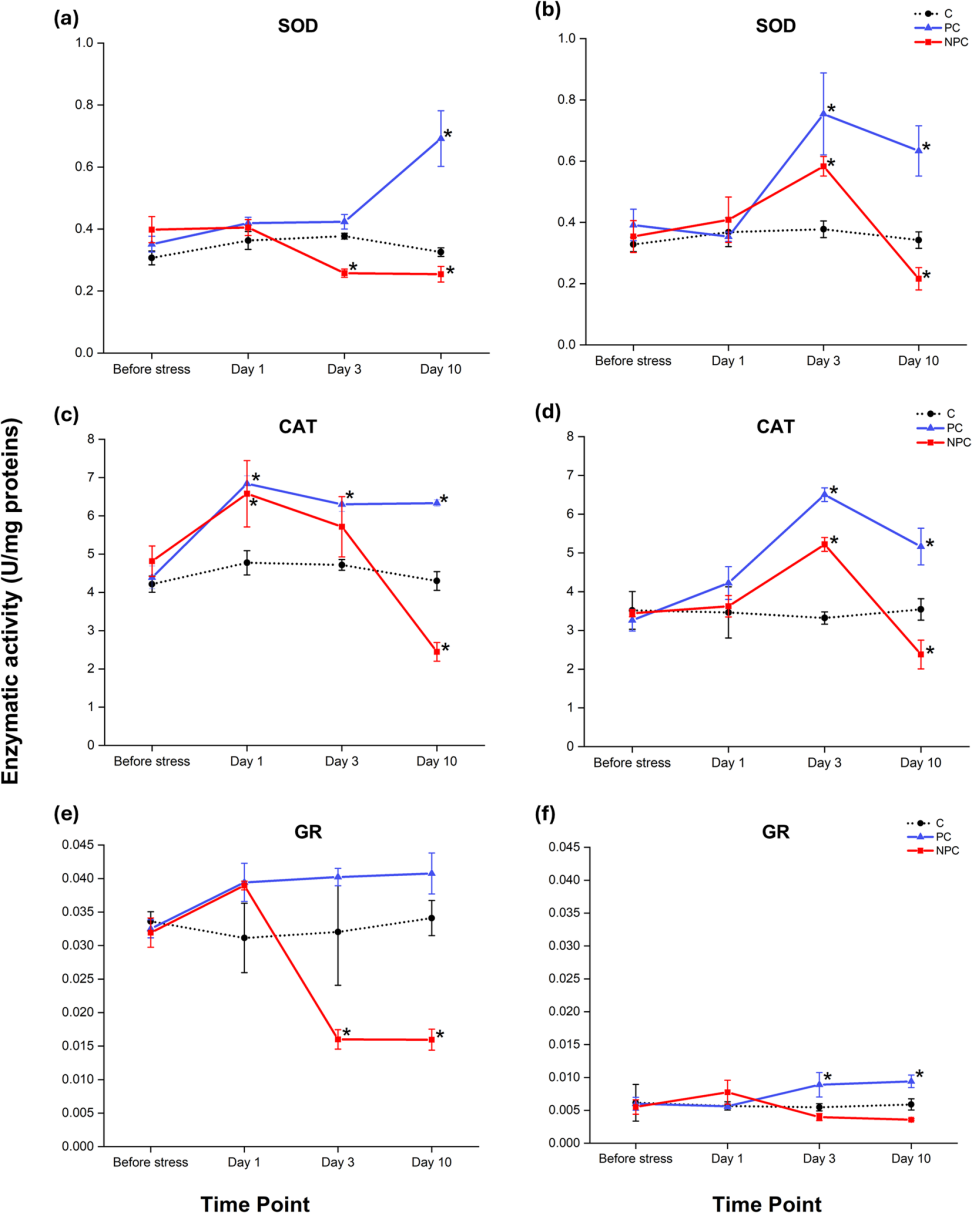
Enzymatic activity of SOD **(a**,** b)**, CAT **(c**,** d)**, and GR **(e**,** f)** in *P. damicornis* and *S. pistillata* under three different treatments (C, PC, NPC) at specific time intervals (Before stress, Day 1, Day 3, Day 10). Data are expressed as units per milligram of protein (U/mg protein) and are presented as means ± SEM. Asterisks indicate statistically significant differences (*p* < 0.05) from the control group (C) at the corresponding time points, as determined by the two-factor univariate PERMANOVA followed by pairwise comparisons. Error bars represent the standard error of the mean. Full PERMANOVA pairwise comparisons are provided in Table S2 and S4 (Supplementary Information).

To assess the antioxidant capacity of corals in countering ROS produced during heat stress, the activities of three key antioxidant enzymes, superoxide dismutase (SOD), catalase (CAT), and glutathione reductase (GR) were measured (Fig. 4). Similar patterns were observed in both coral species. The activity of the three investigated antioxidant enzymes remained relatively constant in the control groups throughout the experiment.

#### Superoxide dismutase activity

In *P. damicornis*, the different treatments and their interaction at the different sampling time points significantly affected the SOD activity (Table S1). SOD activity remained stable in preconditioned corals until Day 3, increasing by Day 10 (Fig. 4a). In contrast, non-preconditioned corals showed no significant changes in SOD activity compared to the control or preconditioned corals on Day 1 (Table S2). However, by Day 3, SOD activity significantly decreased in non-preconditioned corals relative to controls, and this reduced level persisted through Day 10 (Table S2).

In *S. pistillata*, all the investigated factors and their interaction significantly changed the activity of SOD (Table S3). SOD activity was comparable across all treatments, with no statistically significant differences on Day 1 of acute heat stress compared to control group (Fig. 4b). On Day 3, however, SOD activity increased in preconditioned corals and it was higher compared to both non-preconditioned and control groups (Table S4). By Day 10, SOD activity in preconditioned corals remained stable, while in non-preconditioned corals it significantly dropped below the control levels.

#### Catalase activity

In both coral species all the investigated factors and their interaction significantly affected the activity of CAT (Tables S1 and S3). In *P. damicornis*, both preconditioned and non-preconditioned corals exhibited a significant increase in CAT activity compared to controls on Day 1 (Table S2). On Day 3, CAT activity was sustained in preconditioned corals, while in non-preconditioned corals CAT activity began to decrease (Fig. 4c). Preconditioned corals continued to maintain higher CAT activity level through Day 10, whereas CAT activity in non-preconditioned corals continued to decline and was significantly lower compared to both preconditioned and control corals (Table S2).

Similar patterns were observed in *S. pistillata* (Fig. 4d). CAT activity was higher in preconditioned corals compared to non-preconditioned and controls on Day 1, although the difference was not statistically significant. This elevated activity persisted, and by Day 3 and Day 10, CAT activity was significantly higher than in non-preconditioned and control corals (Table S4). In non-preconditioned corals, CAT activity initially increased following acute heat stress and ultimately decreased below control levels by Day 10.

#### Glutathione reductase activity

In *P. damicornis*, GR activity showed significant changes across the different treatments and sampling time points analysed (Table S1). Both preconditioned and non-preconditioned *P. damicornis* corals exhibited a similar, increase in GR activity compared to controls after Day 1, although it was not statistically significant (Fig. 4e; Table S2). GR activity remained elevated in preconditioned corals from Day 3 through Day 10 compared to both non-preconditioned and control corals. In contrast, non-preconditioned corals showed a significant decrease in GR activity after Day 3, with activity remaining below that of both control and preconditioned corals by Day 10 (Table S2).

In *S. pistillata*, significant differences in GR activity were detected only at different time points (Table S2). In particular, in preconditioned *S. pistillata* corals, GR increased following acute heat stress and remained elevated compared to both non-preconditioned and control corals from Day 3 through Day 10 (Fig. 4f; Table S4). In non-preconditioned corals, GR activity was not significantly different from controls throughout the experiment (Table S4).

### Preconditioned corals show lower lipid peroxidation levels

**Fig. 5:**
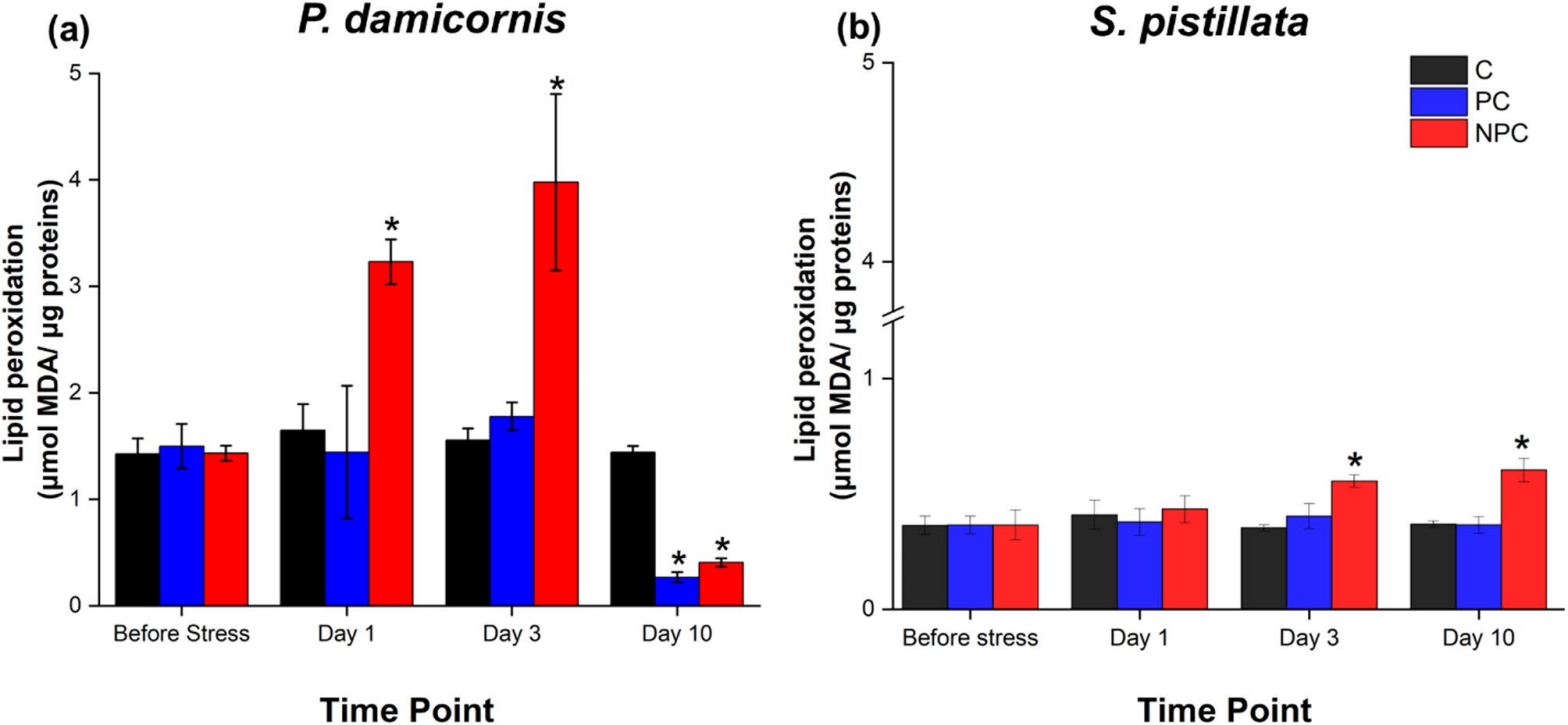
Levels of lipid peroxidation in *P. damicornis* (**A**) and *S. pistillata* (**B**) under three different treatments (C, PC, NPC) at specific time intervals (Before stress, Day 1, Day 3, Day 10). Data are expressed as µmol of MDA per µg of proteins, and as mean ± SEM. Asterisks indicate statistically significant differences (*p* < 0.05) from the control group (**C**) at the corresponding time points, as determined by the two-factor univariate PERMANOVA followed by pairwise comparisons. Error bars represent the standard error of the mean. Full PERMANOVA pairwise comparisons are provided in Table S2 and S4 (Supplementary Information).

To assess oxidative damage within coral cells, lipid peroxidation (LPO) was quantified by measuring malondialdehyde (MDA) content (Fig. 5). In the control groups of each species, MDA levels remained relatively constant throughout the experimental period, indicating the physiological baseline. In *P. damicornis*, MDA concentration showed significant change between treatments, sampling time points and following the interaction of both factors (Table S1). In particular, non-preconditioned *P. damicornis* colonies exhibited a marked increase in MDA levels compared to both control and preconditioned corals as early as Day 1 following the onset of acute heat stress (Fig. 5a; Table S2). This increase persisted, with MDA levels rising to 2.24-fold higher than those observed in preconditioned corals by Day 3 (Table S2). After Day 10, MDA levels in non-preconditioned corals declined to values below those of the control group. In contrast, preconditioned corals maintained MDA levels comparable to the controls on both Day 1 and Day 3 (Table S2). By Day 10, MDA levels in preconditioned corals had decreased to levels below those observed in the control group (Table S2).

In *S. pistillata*, only the different treatments significantly affected MDA levels (Table S2). In non-preconditioned *S. pistillata* colonies increased progressively as the acute heat stress continued, reaching 1.58-fold higher than control levels by Day 3 and 1.63-fold higher by Day 10 (Fig. 5b; Table S4). In contrast, preconditioned corals maintained MDA levels similar to those of the control group throughout the experiment, including on Day 10 (Table S4).

### Preconditioned corals exhibited higher levels of Hsp70 protein and delayed upregulation of the *hsp70* gene following thermal stress.

**Fig. 6:**
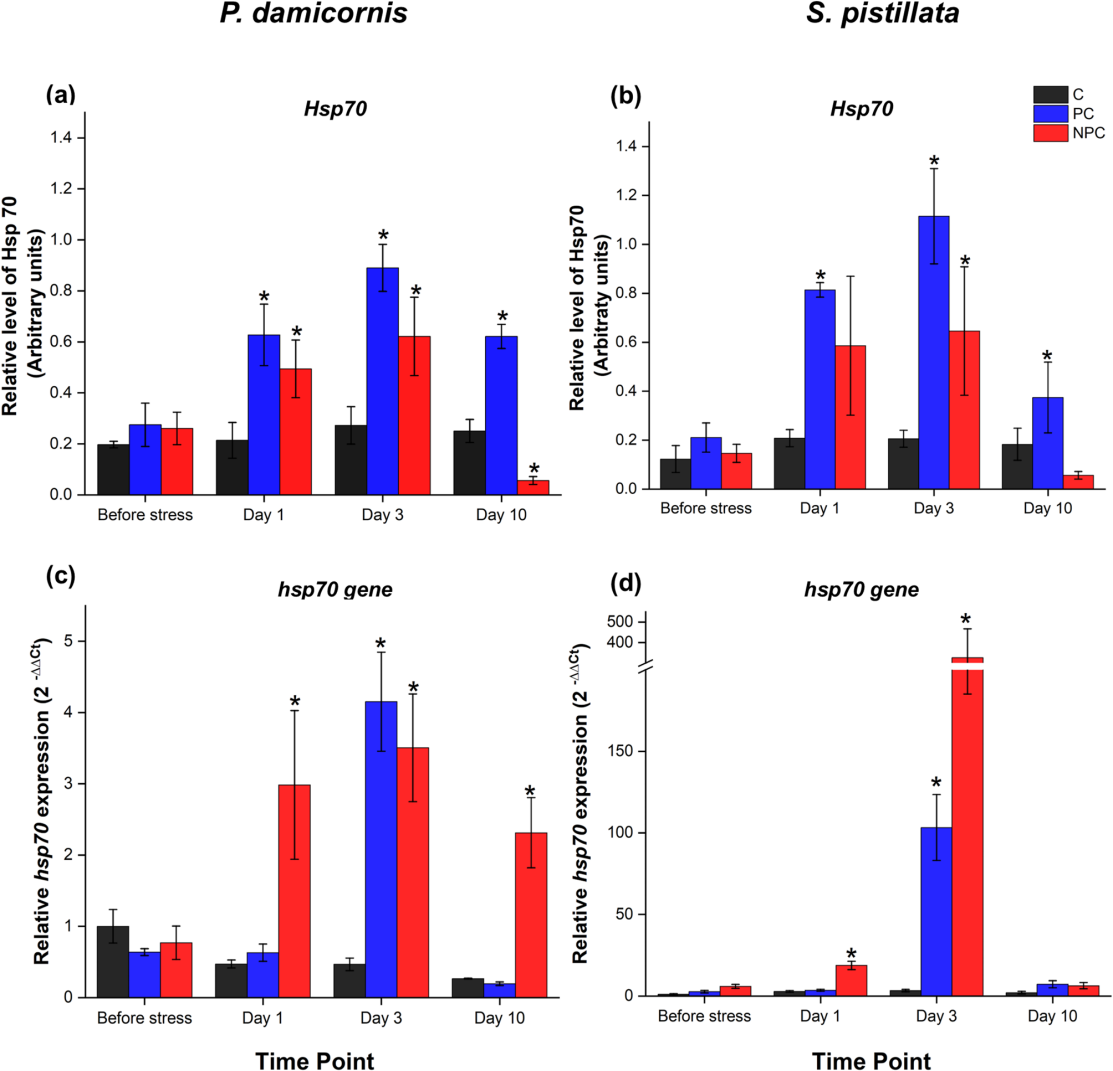
Expression of Hsp70 protein and *hsp70* gene in (**a**,**c**) *Pocillopora damicornis* and (**b**,**d**) *Stylophora pistillata* under three different treatments (C, PC, NPC) at specific time intervals (Before stress, Day 1, Day 3, Day 10). Panels (**a**,**b**) show Hsp70 protein levels, expressed in arbitrary units. Panels (**c**,**d**) show *hsp70* gene expression, normalised and expressed as 2^–ΔΔCt^ relative to the “Control group” at the “Before Stress” time point. Asterisks indicate statistically significant differences (*p* < 0.05) from the control group (C) at the corresponding time points, as determined by the two-factors univariate PERMANOVA followed by pairwise comparisons. Error bars represent the standard error of the mean. Full PERMANOVA pairwise comparisons are provided in Table S2 and S4 (Supplementary Information).

Hsp70, a crucial molecular chaperone, plays a key role in cytoprotection by maintaining protein integrity under heat stress induced bleaching^37,38^. *P. damicornis* nubbins modulated Hsp70 differently, both between and within the different treatments and the sampling times (Table S1). In particular, Hsp70 levels remained stable in the control group throughout the experiment, indicating consistent baseline modulation. Before acute heat stress, Hsp70 levels were similar across all treatment groups (Fig. 6a). However, on Day 1, preconditioned corals showed a significant increase in Hsp70 levels compared to control group (Table S2). Notably, Hsp70 levels in preconditioned corals were even higher (1.5-fold) on Day 3 compared to non-preconditioned corals (Table S2), indicating a sustained or enhanced protective response. Although Hsp70 levels decreased by Day 10, they remained significantly higher than in both control and non-preconditioned corals (Table S2).

In non-preconditioned corals, Hsp70 levels were significantly higher compared to controls at both Day 1 and Day 3 after heat stress, but lower than those observed in preconditioned corals (Table S2). After Day 10, however, Hsp70 levels in non-preconditioned corals decreased considerably, dropping to levels lower than those of the control samples (Table S2).

In *S. pistillata*, the expression of Hsp70 was significantly affected by treatments and sampling time points (Table S3), and a similar pattern of expression to that of *P. damicornis* was observed (Fig. 6b). Hsp70 levels remained stable in the control group throughout the experiment. Preconditioned corals showed higher Hsp70 levels after acute heat stress, particularly on Day 3 (1.7-fold) and Day 10 (6.7-fold) compared to non-preconditioned corals (Table S4). By Day 10, no statistical difference in Hsp70 levels was observed between non-preconditioned and control corals. Additionally, following stress, preconditioned corals of both species had a slower rate of Hsp70 downregulation over time (Table S4).

To understand the transcriptional responses associated with the observed Hsp70 protein dynamics, we investigated the corresponding expression patterns at the gene expression level. A similar trend in *hsp70* gene expression was observed across both species, which showed significant changes in *hsp70* gene levels based on treatments, sampling time points and the interaction of both factors (Tables S1 and S3). In control groups of both species, *hsp70* expression remained stable throughout the experimental period (Fig. 6).

In *P. damicornis*, non-preconditioned corals showed a significant upregulation of *hsp70* expression after Day 1 of acute heat stress (2.99-fold), with further increase observed after Day 3 (3.5-fold; Table S2). In contrast, preconditioned corals exhibited no change in *hsp70* expression after Day 1 but a 4.2-fold increase after Day 3 was observed (Fig. 6c). No significant differences were detected between preconditioned and non-preconditioned corals on Day 3 (Table S2). By Day 10, *hsp70* expression in non-preconditioned corals remained elevated (2.31-fold), while it decreased in preconditioned corals, returning to levels comparable to those of the controls (Table S2).

In *S. pistillata*, non-preconditioned corals exhibited a significant increase in *hsp70* expression, reaching an 18.7-fold rise after Day 1 (Fig. 6d; Table S4). This increase continued, reaching a 326-fold rise after Day 3, before returning to baseline levels comparable to the control group after Day 10. In contrast, preconditioned corals showed a minor, non-significant increase in *hsp70* expression after Day 1 (Table S4). However, after Day 3, *hsp70* expression in preconditioned nubbins was significantly upregulated to 103-fold, though still lower than in non-preconditioned corals (Table S4). By Day 10, *hsp70* expression in preconditioned corals decreased, aligning with levels observed in other treatments.

### A cascade of protective responses delaying bleaching

**Fig. 7:**
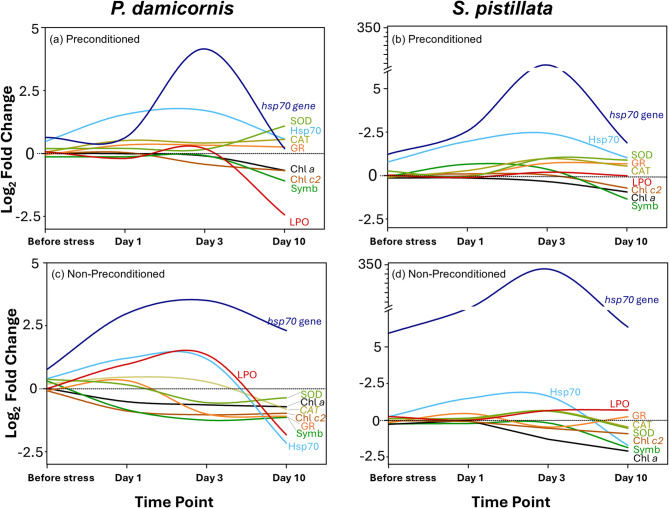
Visual comparison of multi-biomarker responses in preconditioned and non-preconditioned colonies of *P. damicornis* (**a**,**c**) and *S. pistillata* (**b**,**d**) under thermal stress. Fold changes were calculated relative to controls at each time point and presented as log_2_ fold change.

In both species, preconditioned corals displayed earlier and/or sustained activation of protective mechanisms, notably elevated antioxidant enzyme activity (SOD, CAT, GR) and Hsp70 protein expression (Fig. 7). This response helped limit oxidative stress and supported longer-term cellular homeostasis, resulting in prolonged retention of chlorophyll pigments and Symbiodiniaceae. Notably, in *P. damicornis*, oxidative damage (LPO) remained low in preconditioned corals, whereas non-preconditioned colonies showed a pronounced spike in LPO. In *S. pistillata*, LPO remained relatively stable in preconditioned colonies, while it gradually increased under stress in non-preconditioned ones. In both species, *hsp70* gene expression increased rapidly in non-preconditioned corals following stress, whereas in preconditioned corals, *hsp70* expression was delayed, suggesting an enhanced capacity to cope with thermal stress.

## Discussion

This study investigated the effects of thermal preconditioning in two of the most used coral species in coral restoration using a multi-biomarker approach. Overall, our findings suggest that thermal preconditioning increases the thermal tolerance of the studied corals, bringing further evidence of its potential utility in improving the thermal tolerance of these species in coral reef restoration programmes. In particular, our results revealed that in both species, thermally preconditioned corals exhibited delayed bleaching, as evidenced by prolonged retention of chlorophyll *a* and *c2*, as well as Symbiodiniaceae. These preconditioned corals maintained sustained enzymatic activity of key antioxidant enzymes (SOD, CAT, GR) and showed elevated levels of Hsp70 protein as well as lower post-stress Hsp70 downregulation, indicating enhanced protection against oxidative stress and better maintenance of cellular homeostasis. Additionally, these corals exhibited lower levels of lipid peroxidation (LPO), suggesting reduced oxidative damage within their cells, and a lower expression of the *hsp70* gene, indicative of a lower immediate stress response. Overall, PERMANOVA analyses supported these patterns, revealing significant effects of preconditioning and time on the measured physiological and molecular biomarkers.

This delay in bleaching among preconditioned *P. damicornis* and *S. pistillata* corals highlights the effectiveness of thermal preconditioning. The prolonged retention of chlorophyll *a*, chlorophyll *c2*, and Symbiodiniaceae density implies that preconditioned corals were able to maintain their vital symbiotic relationship with their Symbiodiniaceae and preventing bleaching for a longer period under thermal stress. This enhanced capacity to retain Symbiodiniaceae and photosynthetic pigments suggests a robust acclimation response that helps mitigate the adverse effects of elevated temperatures, leading to improved survival under thermal stress. Similar studies such as Bellantuono et al. (2012a, b) and DeMerlis et al. (2022), found that preconditioned *A. millepora* and *A. cervicornis* colonies maintained high Symbiodiniaceae density and delayed severe stress responses, suggesting a protective effect. Similarly, Bay and Palumbi (2015) reported extended chlorophyll *a* retention in preconditioned *A. nana*, while Majerová et al. (2012) observed that *P. acuta* exposed to sublethal temperatures exhibited slower bleaching compared to non-preconditioned corals. Conversely, other studies, including Middlebrook et al. (2012) and Gibbin et al. (2018), found that preconditioning had limited or negligible effects on thermal tolerance in *A. millepora* and *P. damicornis*, respectively. These inconsistent outcomes may be due to differences in experimental protocols across studies.

Moreover, our findings revealed that the effectiveness of thermal preconditioning varies among coral species, partly due to their inherent differences in naive thermal tolerance. In *P. damicornis*, non-preconditioned corals exhibited bleaching on Day 1, while preconditioned corals showed a 9-day delay, bleaching only on Day 10. In contrast, *S. pistillata* non-preconditioned corals, which already exhibited greater naive thermal tolerance, began to bleach on Day 3, while preconditioned *S. pistillata* corals experienced bleaching on Day 10, reflecting a 7-day delay. This suggests that, while preconditioning improved thermal tolerance in both species, *P. damicornis* had a more pronounced response to preconditioning. This disparity underscores the species-specific nature of coral thermotolerance^39^ and highlights the challenge of applying general conclusions across different coral taxa.

In general, heat shock proteins are typically basally expressed in corals but are upregulated in response to thermal stress, playing a key role in stress tolerance. When stress exceeds a certain threshold, Hsp expression can be downregulated, potentially leading to cellular damage and reduced resilience. Thermotolerant corals, however, have been found to maintain higher levels of heat shock proteins compared to more thermally susceptible colonies, suggesting a link between heat shock proteins expression and stress resilience. Thermally preconditioned corals exhibited higher levels of Hsp70 protein compared to non-preconditioned corals in both species. This observation aligns with numerous studies reporting elevated Hsp70 levels in bleaching-tolerant corals compared to those more susceptible to bleaching^37,40–42^. Notably, Louis et al. (2020) demonstrated that corals naturally thermally preconditioned in an environment with greater temperature variability had increased Hsp70 levels. In addition, preconditioned corals exhibited a slower rate of Hsp70 downregulation following acute heat stress. This is an indicator of enhanced thermal tolerance, as observed by Seveso et al. 2014, 2016, 2020^37,43,44^, who reported that more thermotolerant corals consistently show a delayed downregulation of heat shock proteins after stress. These findings strongly suggest that preconditioning effectively enhances the thermal tolerance of corals. The role of Hsp70 in heat tolerance is well-documented; as a molecular chaperone, Hsp70 helps prevent protein denaturation and loss of functional conformation during heat stress, thereby maintaining cellular integrity and homeostasis^45–47^. Furthermore, Hsp70 is essential in corals for preserving protein homeostasis during oxidative stress induced by different stressors^38,48,49^. These findings underscore the importance of Hsp70 upregulation as a key factor contributing to enhanced thermal tolerance in corals.

In contrast, our *hsp70* gene results exposed complexities not captured by protein expression analysis. Notably, no significant changes in gene expression were detected in both species during the preconditioning phase, which is consistent with Bay and Palumbi (2015) who also found no changes during preconditioning. Similarly, Seneca and Palumbi (2015) reported transient increases in the expression of 19 *hsp* genes shortly after heat stress, with only a few genes remaining elevated after extended periods (> 20 h). This may suggest that the three-day recovery and the sampling period used in our study might have been too long to capture the initial gene upregulation following sub-lethal stress. Additionally, *hsp70* in preconditioned corals showed a muted reaction to heat stress, with non-preconditioned corals exhibiting a faster and more pronounced response. This observation aligns with Bay and Palumbi (2015), who noted that preconditioned corals demonstrate a less pronounced stress response. In this context, Barshis et al. (2013) proposed that reduced stress responses in tolerant corals might be due to higher constitutive levels of stress-indicator proteins, or that such reduced responses could signify resilience rather than causation. For instance, more heat-tolerant corals could have experienced lower physiological stress, leading to smaller expression changes in stress-indicator genes. This perspective is also supported by Louis et al. 2017^41^, who identified *hsp70* gene as an early responder to stress, reinforcing the complex relationship between stress responses and thermal tolerance.

While Hsp70 protein levels and *hsp70* gene expression offered important perspectives, the analysis of the antioxidant enzyme activity provides additional insights into how corals improve their tolerance following thermal preconditioning. It is known that under thermal stress, the photosynthetic Symbiodiniaceae produce reactive oxygen species (ROS) at levels that exceed the host’s cellular capacity, leading to cellular damage, necrosis, or detachment^48,50^. Excessive ROS triggers signalling pathways that result in the degradation or expulsion of the symbionts, causing coral bleaching and, under prolonged stress, coral death due to starvation^51,52^. To prevent bleaching, corals increase the activity of antioxidant enzymes as SOD, CAT, and GR to combat oxidative stress^53^. Enhanced antioxidant enzyme activity in response to thermal stress has thus been extensively documented in corals^48,54–57^. In our study, consistent changes in three key antioxidant enzymes SOD, CAT and GR in both *P. damicornis* and *S. pistillata* following heat stress was observed. In particular, antioxidant enzymatic activities increased after just one day of heat stress and remained elevated in preconditioned corals. In contrast, non-preconditioned corals initially showed increased levels of SOD, CAT, and GR, but these levels decreased over time, eventually falling below control levels. Therefore, we suggest that non-preconditioned corals were unable to sustain or further boost their antioxidant defences as heat stress persisted. Hawkins and Warner (2017) found no significant differences in SOD activity between preconditioned and non-preconditioned sea anemones and highlighted the need for careful interpretation due to small sample sizes. In contrast, Majerová and Drury (2022) observed that preconditioned corals, with greater thermal tolerance and reduced bleaching rates, exhibited higher GR activity compared to non-preconditioned corals.

 When ROS levels exceed the host’s ability to maintain cellular homeostasis, they cause various forms of cellular damage, as a result of lipid peroxidation, protein oxidation, enzyme inhibition, and damage to nucleic acids^58–60^. To assess oxidative damage and dysfunction in coral cells especially due to thermal stress, lipid peroxidation (LPO) was commonly used as a stress biomarker^38,48,55,61^, quantified by measuring malondialdehyde content. MDA is widely used as a marker for lipid peroxidation because it is one of the most prevalent and stable byproducts formed during the degradation of polyunsaturated fatty acids (PUFAs) in cell membranes under oxidative stress^62^. Therefore, higher MDA levels indicate greater membrane degradation. Our results showed significantly higher lipid peroxidation in non-preconditioned corals compared to preconditioned corals. Specifically, LPO level in non-preconditioned corals increased with the duration of thermal stress, whereas LPO level in preconditioned corals remained comparable to those in control samples. These results suggest that in both species preconditioned corals experienced less oxidative damage than their non-preconditioned counterparts. This reduced oxidative damage in preconditioned corals may be attributed to their higher levels of antioxidant enzymes and Hsp70, which likely contribute to enhanced cellular protection and stress resilience. Our results are consistent with those of Huang et al. (2024) who observed decreased LPO and caspase-3 activity in acclimated corals, suggesting a delayed apoptotic process that enables corals to withstand extreme heat stress. They suggested that acclimated corals experience less reactive oxygen species (ROS) damage compared to unacclimated corals.

Our findings contribute to a broader understanding of stress response cascades and stress memory in corals. We observed that thermal preconditioning may promote initial physiological adjustments and some degree of molecular preparedness, as preconditioned corals showed elevated Hsp70 expression and antioxidant enzyme activity during acute thermal exposure. These changes appeared to enhance cellular protection and support homeostasis. As a result, preconditioned corals experienced reduced oxidative stress, as indicated by better retention of chlorophyll and symbionts. This is consistent with previous studies showing that preconditioning can mitigate oxidative damage through enhanced antioxidant responses^30,63^. Notably, Majerová & Drury (2022) also reported that non-preconditioned corals accumulated DNA damage (oxidized guanine species, 8-OHdG), whereas preconditioned corals did not. The improved protective state appeared to help preconditioned corals better tolerate subsequent severe thermal stress, with a delayed onset and reduced magnitude of stress responses, as indicated by lower *hsp70* gene expression and diminished oxidative damage during later stress events. Moreover, preconditioned corals maintained symbiosis stability for longer and showed slower rates of bleaching compared to non-preconditioned corals.

In a similar multibiomarker study, Gardner et al. (2017)suggested a stress-response cascade in *S. pistillata* following thermal stress, with early antioxidant activation followed by declines in chlorophyll content, symbiont density, and photosynthetic performance, ultimately leading to bleaching. In contrast, the present study shows that preconditioning shifts this cascade by delaying bleaching onset and sustaining higher chlorophyll and symbiont densities. Antioxidant responses (SOD, CAT, GR) also remained elevated for a longer duration in preconditioned corals, indicating extended protection against oxidative stress. These findings suggest that preconditioning modifies the typical reaction sequence.

Overall, our results support previous research suggesting that thermal priming can enhance thermal tolerance by allowing the coral’s cellular machinery to respond more effectively to repeated stress, potentially improving resilience to future stressors^26,29,30,64^. However, the efficiency of thermal preconditioning in corals depends on the specific temperature regime applied. Ferrara et al. (2025) found that variable thermal regimes slightly outperformed stable ones, improving thermal tolerance and recovery. In contrast, Drury et al. (2022) showed that only constant high and pulse exposures enhanced thermal tolerance in *Montipora capitata*. Both studies highlight the importance of the intensity, duration, and variability of preconditioning treatments. Additionally, species-specific traits and genotypic differences significantly influence the outcomes of thermal acclimation.

In addition to controlled experimental preconditioning, naturally occurring thermal fluctuations may similarly influence coral stress responses in the field. Degree Heating Weeks (DHW) are widely used to predict bleaching, yet our results suggest that moderate thermal anomalies (e.g., DHW < 4 °C-weeks) preceding more severe heat events (DHW > 8 °C-weeks) could promote physiological adjustments and molecular preparedness similar to experimental preconditioning. This indicates that corals experiencing mild thermal stress may enter subsequent heatwaves with increased level of thermal protection. Integrating such natural thermal histories and potential stress memory effects into predictive bleaching models could improve their precision and better reflect coral responses under fluctuating environmental conditions.

In conclusion, our study offers a comprehensive analysis of how thermal preconditioning could possibly enhance coral tolerance to heat stress, at both molecular and cellular levels. Preconditioned corals exhibited reduced oxidative stress, as shown by improved retention of chlorophyll and symbionts. This increased bleaching tolerance is linked to enhanced thermal protection and maintenance of the cellular homeostasis through elevated levels of Hsp70 expression and antioxidant enzyme activity. Consequently, preconditioned corals showed fewer signs of cellular stress, such as lower *hsp70* gene expression and reduced oxidative damage. *P. damicornis* displayed a more pronounced response to thermal preconditioning, highlighting species-specific differences. The use of a multi-biomarker approach could be pivotal in revealing the complex molecular adaptations triggered by thermal preconditioning, offering new insights beyond studies that have typically focused on isolated stress response components. This study highlights the intricate and multifaceted nature of coral tolerance and suggests directions for future studies, including exploring additional molecular pathways, testing other coral species, refining preconditioning protocols for greater effectiveness, and validating these findings in natural environments. Overall, our study confirms the effectiveness of thermal preconditioning in enhancing coral tolerance to thermal stress, positioning it as a vital technique to be incorporated into coral restoration efforts in response to rising ocean temperatures driven by global warming.

## Methods

### Experimental setup

#### Coral sample preparation and acclimation

At the Aquarium of Genoa (Italy), 96 small coral nubbins (~ 10 cm each) of *P. damicornis* and *S. pistillata* were fragmented from eight large mother colonies of each species. To ensure consistency during the experiment and avoid variations in results based on the most abundant genus of microalgal endosymbionts (Symbiodiniaceae) hosted by the corals, the genus of Symbiodiniaceae in the mother colonies was determined prior to the experiment. Specifically, DNA was extracted from the eight mother colonies of each species with the DNeasy Blood and Tissue Kit (Qiagen, Germany), a portion of the ITS2 region was amplified using the primers SYM_VAR_5.8S2 and SYM_VAR_REV, as described in^65,66^, and PCR products were sequenced with an ABI 3730xl DNA Analyser (Applied Biosystems, USA). The obtained sequences were compared to the NCBI database using the Basic Local Alignment Search Tool (BLAST) and were finally deposited in GenBank with the accession numbers PP264470-PP264485. Nubbins were sourced from mother colonies hosting the same Symbiodiniaceae genus: *P. damicornis* nubbins from colonies predominantly hosting genus *Cladocopium* (GenBank accession numbers: PP264470-PP264477) and *S. pistillata* nubbins from colonies predominantly hosting genus *Symbiodinium* (GenBank accession numbers: PP264478-PP264485). All 96 nubbins were acclimated in the same tank for two weeks at the control temperature of 25 °C and light: dark period of 11:13 h. The irradiation in acclimation tank was equal to 250 PAR (m^−2^ s^−1^ µmol photons). Technical details of the flow-through aquarium setup, based on Isa et al. (2024), are reported in Supplementary Material S1.

#### Temperature treatments and coral sampling

The corals used in the present study were cultivated at an optimum temperature of 25 °C. Prior to the experiment, preliminary trials were conducted to determine the bleaching temperature thresholds of the two species, both of which were found to bleach at 32 °C. Following the acclimation period, nubbins were randomly distributed across three distinct temperature treatments in duplicated aquaria (Fig.S1). In the control (C) tanks, the temperature was maintained consistently at control temperature (25 °C) throughout the entire duration of the experiment (Fig. 1). In the preconditioning (PC) tanks, the temperature was gradually increased to 28 °C at a rate of + 1 °C per day and maintained for seven days. This preconditioning temperature which is + 3 °C above the control temperature, was selected based on the methodology described by Majerová et al. 2021^63^. Following the preconditioning phase, the temperature in the preconditioning tanks was gradually decreased by −1 °C per day until it returned to 25 °C, where it was maintained for an additional five days for recovery. Subsequently, the temperature was increased by + 1 °C per day until it reached 32 °C, which corresponds to the experimentally determined bleaching threshold of the samples. This temperature was maintained for ten days. In this study, we refer to this phase as the acute stress treatment (as it represents the bleaching temperature of the samples). In the non-preconditioned (NPC) tanks, the temperature was increased directly from 25 °C to 32 °C at a rate of + 1 °C per day, without a preconditioning phase. Upon reaching 32 °C, this temperature in the NPC tanks was maintained for ten days. Each temperature treatment consisted of two duplicated tanks, each containing 16 nubbins of the same species, for a total of 32 nubbins per treatment per each species.

Degree Heating Week (DHW) was calculated following the approach described by Ferrara et al. (2025). Cumulative heat stress was quantified by summing daily temperature anomalies exceeding the bleaching threshold, defined as 1 °C above the maximum monthly mean (MMM). For this experiment, the MMM was considered 25 °C, based on the stable-ambient baseline temperature of the facility where the corals have been cultured for over a decade. Accordingly, the bleaching threshold was set at 26 °C. During the preconditioning phase, corals were exposed to a cumulative heat stress of 2.43 °C-weeks (DHW < 4 °C-weeks is below the bleaching threshold). During the subsequent acute thermal stress phase, the cumulative heat exposure reached 11.57 °C-weeks (DHW > 8 °C-weeks corresponds to severe thermal stress, expected to cause widespread coral bleaching and potential mortality).

Four different time points were set during the experiment: (1) prior to the initiation of the acute heat stress ramping phase (before stress), (2) one day after reaching the maximum stress temperature of 32 °C, and (3) three days, and (4) ten days following the onset of acute heat stress (Fig. 1). Before sampling at each time point, coral fragments were quickly photographed out of water against a white background using the same camera and consistent lighting conditions (Fig. 2), then immediately returned to their respective tanks to minimise stress. Bleaching was considered present when at least 7 out of 8 fragments within a treatment group exhibited visible paling or loss of pigmentation. At each time point, four random nubbins per duplicated tank were collected (*n* = 8 per treatment), with concurrent sampling performed in the control tanks matching the PC and NPC treatments. The collected nubbins were fragmented in half, with one half stored at −20 °C for bleaching assessments and the other half immediately snap-frozen in liquid nitrogen and stored at −80 °C for molecular biology analyses.

### Quantification of bleaching

#### Chlorophyll *a* and *c2* quantification

Coral tissue was blasted off from coral fragments (stored at –20 °C) using filtered compressed air^67^. Chlorophyll was extracted from tissue following Isa et al. 2024 and Louis et al. (2016). Absorbance of acetone extract was measured at 630, 663, and 750 nm. Chlorophyll concentration was calculated using dinoflagellate-specific equations^68^ and normalised to coral surface area. The surface area of the coral fragments was determining using the paraffin wax dipping method, following the procedure outlined by Isa et al. (2024) and Veal et al. (2010). The detailed methodology is reported in Supplementary Material S2.

#### Symbiodiniaceae density

Symbiodiniaceae cells were counted from six independent hemocytometer (Improved Neubauer) counts, using an optical microscope (Leica Company, France). Cell density was calculated from the surface area of respective fragments^69,70^.

### Analysis of the enzymatic activities

#### Protein extraction

Approximately 1 g of frozen coral fragment was cut from each nubbin stored at −80 °C. Each coral fragment was ground using a pre-chilled mortar and pestle and homogenised in 750 µl lysis buffer (Tris–HCl 50 mM, pH 7.4, NaCl 150 mM, glycerol 10%, NP40 detergent 1%, EDTA 5 mM) containing 1 mM phenylmethylsulfonylfluoride (Sigma-Aldrich). Total protein content was extracted and quantified as per Montalbetti et al. (2021). The detailed methodology is reported in Supplementary Material S3.

#### Antioxidant enzyme activity assay

Superoxide dismutase activity (SOD) was assessed according to Vance et al. (1972) and Montalbetti et al. (2021). Catalase (CAT) activity was assessed by considering the peroxidase function of the enzyme. The method is based on the breakdown of hydrogen peroxide (H_2_O_2_) by the enzyme, as previously described in Bergmeyer et al. (1983). The enzymatic assay of glutathione reductase (GR) was performed according to Wang et al. (2001). Results are expressed as units (U) of enzyme per mg of proteins. The detailed methodology is reported in Supplementary Material S3.

### Lipid peroxidation

Approximately 1 g of frozen coral fragment was cut from each nubbins stored at −80 °C. Each coral fragment was ground in a pre-chilled mortar and pestle and homogenised in 1 ml of 20 mM phosphate buffer, pH 7.4. To prevent sample oxidation, 10 µl of 0.5 M butylated hydroxytoluene in acetonitrile were added to 1 ml of tissue homogenate. Lipid peroxidation levels were measured by assessing malondialdehyde (MDA) concentrations following Montalbetti et al. (2021) and using the commercially available MDA assay kit (Bioxytech LPO-586, Oxis International, USA). The detailed methodology is reported in Supplementary Material S4.

### Analysis of the Hsp70 expression

#### Protein extract Preparation and western blot analysis

Approximately 1 g of frozen coral fragment was cut from each nubbin stored at −80 °C. Each fragment was then ground using a pre-chilled mortar and pestle and homogenised in SDS-buffer (0.0625 M Tris–HCl, pH 6.8, 10% glycerol, 2.3% SDS, 5% 2-mercaptoethanol) containing 1 mM phenylmethylsulfonylfluoride (Sigma-Aldrich), and complete EDTA-free protease inhibitors cocktail (Roche Diagnostic). Western Blot analysis was carried out as previously described by Seveso et al. (2012; 2020). The detailed methodology is reported in Supplementary Material S5.

### Gene expression analysis

Total RNA was extracted as described in Isa et al. (2024). Briefly, for each sample, a coral fragment (0.3–0.5 g) was cut from each nubbin stored at −80 °C. Coral tissue was blasted off the coral fragment in a pre-cooled mortar, using filtered compressed air for a maximum of 3 min^67^. RNA extractions were then continued using the Qiagen RNA Mini kit (Qiagen, Germany) following the manufacturer’s instructions for purification of total RNA from animal tissues. DNA contamination was removed using the DNase I Set (Zymo Research, USA) in combination with the RNA Clean & Concentrator-25 kit (Zymo Research) according to the manufacturer’s protocol. RNA quality was checked by examining with gel electrophoresis for clear, sharp bands of ribosomal RNAs. RNA concentration was estimated using Qubit (RNA Broad Range Assay Kit, Thermo Fisher Scientific). Real-Time qPCR was performed using the QuantiNova SYBR Green RT-PCR Kit (Qiagen, Germany) according to the manufacturer’s instructions. Reactions were performed, in triplicate, and as described in^71^. The detailed methodology is reported in Supplementary Material S6.

The *ef* gene has been widely employed as an internal control in numerous studies due to its consistent expression levels across various experimental stress conditions, including heat stress in *P. damicornis*^71–73^. Similarly, *18 S rRNA* has been validated as a reliable internal control under similar conditions in *S. pistillata*^74^. The sequences of the PCR primers used are detailed in Table 1. Primer efficiency was assessed through a serial dilution (ranging from 1 in 10) of a mixed RNA sample, with efficiencies consistently falling between 0.94 and 1.10. Relative changes in gene expression were quantified using the 2^−ΔΔCt^ method^78^. All calculations were performed in R Studio, and gene expression results are presented as log2 fold-changes relative to the control group prior to the onset of acute stress.

**Table 1:** Primer sequences of GOI and internal control gene used for qPCR analyses. In primer sequence column, F and R indicate the orientation (F: forward; R: reverse).

Species	Gene	Primer Sequence 5’ To 3’	Reference
*P. damicornis*	*hsp70*	F: ATCCAGGCAGCGGTCTTGT	^75^
		R: TCGAGCAGCAGGATATCACTGA	^71,76^
	Elongation Factor (*ef*)	F: CGCTGGCAAAGTGACAAAGG	^72^
		R: CAGACTTGCGATGAAATAGATAGGA	^71,73^
*S. pistillata*	*hsp70*	F: AGGCAACTCTCAACCCAAACA	^77^
		R: AGGCAACTCTCAACCCAAACA	
	* 18 s rRNA*	F: AACGATGCCAACTAGGGATCA	^77^
		R: GGTTTCCCATAAGGTGCCAAA	

### Statistical analyses

To assess whether the duplicate tanks in each treatment group differed significantly (“tank effect”; Seveso et al. 2016), statistical tests were performed. One-way ANOVA was used for normally distributed data, and non-parametric tests for non-normal data. Since the tank effect was not significant for all three treatments, data from duplicated tanks were combined and analysed as a single dataset (*n* = 8 per treatment per time point).

All molecular and physiological biomarkers data were analysed by a two-factor univariate PERMANOVA, using a resemblance matrix based on Euclidean distance and treatments (C, PC, NPC) and sampling time points (Before stress, Day 1, Day 3, Day 10) as fixed factors. The PERMANOVA was run using 9999 permutations with partial sum of squares and unrestricted permutation of raw data to obtain *P* values using the Monte Carlo method.

Following PERMANOVA, pair-wise comparison PERMANOVA tests were conducted for each biomarker analysed to assess significant differences between sampling times in C, PC and NPC treatments, and between treatments Before stress, at Day 1, 3 and 10, using the Monte Carlo method.

Analyses were performed using the statistical package PRIMER-E v.7^88^ with the permutational multivariate analysis of variance (PERMANOVA) + add on^89^.

## Electronic supplementary material

Below is the link to the electronic supplementary material.

Supplementary Material 1

## Data Availability

Sequencing data related to this study are available on NCBI (Accession number: PP264470 - PP264485).

## References

[1] Hoegh-Guldberg, O., Poloczanska, E. S., Skirving, W. & Dove, S. Coral reef ecosystems under climate change and ocean acidification. *Front. Mar. Sci.* **4**, 158. 10.3389/fmars.2017.00158 (2017).

[2] van Hooidonk, R. et al. Local-scale projections of coral reef futures and implications of the Paris agreement. *Sci. Rep.* **6**, 39666. 10.1038/srep39666 (2016). 28000782 10.1038/srep39666 PMC5175274

[3] Duarte, C. M. et al. Rebuilding marine life. *Nature* **580**, 39–51. 10.1038/s41586-020-2146-7 (2020). 32238939 10.1038/s41586-020-2146-7

[4] Boström-Einarsson, L. et al. Coral restoration: A systematic review of current methods, successes, failures, and future directions. *PLoS ONE*. **15**, e0226631 (2020). 31999709 10.1371/journal.pone.0226631 PMC6992220

[5] Suggett, D. J. et al. Restoration as a meaningful aid to ecological recovery of coral reefs. *Npj Ocean. Sustain.* **3**, 20. 10.1038/s44183-024-00056-8 (2024).

[6] Dietzel, A., Bode, M., Connolly, S. R. & Hughes, T. P. The Spatial footprint and patchiness of large-scale disturbances on coral reefs. *Glob Change Biol.* **27**, 4829–4843. 10.1111/gcb.15805 (2021). 10.1111/gcb.15805 34390297

[7] Sully, S. et al. Global patterns of coral bleaching and mortality: implications for conservation and management. *Glob Change Biol.* **28**, 1839–1850. 10.1111/gcb.16083 (2022).

[8] Donner, S., Skirving, W., Little, C., Oppenheimer, M. & Hoegh-Guldberg, O. Global assessment of coral bleaching and required rates of adaptation under climate change. *Glob Change Biol.* **11**, 2251–2265. 10.1111/j.1365-2486.2005.01073.x (2005). 10.1111/j.1365-2486.2005.01073.x 34991281

[9] Donner, S. D., Heron, S. F. & Skirving, W. J. Future scenarios: a review of modelling efforts to predict the future of coral reefs in an era of climate change. In *Coral Bleaching. Ecological Studies* Vol. 205 (eds van Oppen, M. J. H. & Lough, J. M.) (Springer, 2009). 10.1007/978-3-540-69775-6_10.

[10] Hughes, T. et al. Global warming and recurrent mass bleaching of corals. *Sci. Rep.* **7**, 13256. 10.1038/nature21707 (2017). 28300113 10.1038/nature21707

[11] Kleypas, J. et al. Designing a blueprint for coral reef survival. *Biol. Conserv.* **257**, 109107. 10.1016/j.biocon.2021.109107 (2021).

[12] Bay, R. A., Rose, N. H., Logan, C. A. & Palumbi, S. R. Genomic models predict successful coral adaptation if future ocean warming rates are reduced. *Sci. Adv.* **3**, e1701413. 10.1126/sciadv.1701413 (2017). 29109975 10.1126/sciadv.1701413 PMC5665595

[13] Hein, M. Y. et al. *Coral Reef Restoration as a Strategy To Improve Ecosystem services – A Guide To Coral Restoration Methods* (United Nations Environment Programme, 2020).

[14] Putnam, H. M. & Gates, R. D. Preconditioning in the reef-building coral Pocillopora damicornis and the potential for trans-generational acclimatization in coral larvae under future climate change conditions. *J. Exp. Biol.* **218** (15), 2365–2372. 10.1242/jeb.123018 (2015). 26246609 10.1242/jeb.123018

[15] Hawkins, T. D. & Warner, M. E. Warm preconditioning protects against acute heat-induced respiratory dysfunction and delays bleaching in a symbiotic sea anemone. *J. Exp. Biol.* **220**, 969–983. 10.1242/jeb.150391 (2017). 27980125 10.1242/jeb.150391

[16] Dilworth, J. Host genotype and stable differences in algal symbiont communities explain patterns of thermal stress response of *Montipora capitata* following thermal pre-exposure and across multiple bleaching events. *Coral Reefs*. **40**, 151–163. 10.1007/s00338-020-02024-3 (2021).

[17] van Oppen, M. J. H., Oliver, J. K., Putnam, H. M. & Gates, R. D. Building coral reef resilience through assisted evolution. *Proc. Natl. Acad. Sci. USA*. **112**, 2307–2313. 10.1073/pnas.1422301112 (2015). 25646461 10.1073/pnas.1422301112 PMC4345611

[18] Oliver, T. A. & Palumbi, S. R. Do fluctuating temperature environments elevate coral thermal tolerance? *Coral Reefs*. **30**, 429–440. 10.1007/s00338-011-0721-y (2011).

[19] Hackerott, S., Martell, H. A. & Eirin-Lopez, J. M. Coral environmental memory: causes, mechanisms, and consequences for future reefs. *Trends Ecol. Evol.* **36**, 1011–1023. 10.1016/j.tree.2021.06.014 (2021). 34366170 10.1016/j.tree.2021.06.014

[20] Middlebrook, R., Hoegh-Guldberg, O. & Leggat, W. The effect of thermal history on the susceptibility of reef-building corals to thermal stress. *J. Exp. Biol.* **211**, 1050–1056. 10.1242/jeb.013284 (2008). 18344478 10.1242/jeb.013284

[21] Middlebrook, R., Anthony, K. R. N., Hoegh-Guldberg, O. & Dove, S. Thermal priming affects symbiont photosynthesis but does not alter bleaching susceptibility in *Acropora Millepora*. *J. Exp. Mar. Biol. Ecol.* **432**, 64–72 (2012).

[22] Schoepf, V. et al. Stress-resistant corals May not acclimatize to ocean warming but maintain heat tolerance under cooler temperatures. *Nat. Commun.* **10**, 4031. 10.1038/s41467-019-12065-0 (2019). 31530800 10.1038/s41467-019-12065-0 PMC6748961

[23] Roper, C. D., Camp, E. F., Edmondson, J. & Suggett, D. J. Combined impacts of natural recruitment and active propagation for coral population recovery on the great barrier reef. *Mar. Ecol. Prog Ser.* **700**, 95–109. 10.3354/meps14184 (2022).

[24] DeMerlis, A. et al. Pre-exposure to a variable temperature treatment improves the response of *Acropora cervicornis* to acute thermal stress. *Coral Reefs*. **41** (2), 435–445 (2022).

[25] Martell, H. A. Thermal priming and bleaching hormesis in the Staghorn coral, *Acropora cervicornis* (Lamarck, 1816). *J. Exp. Mar. Biol. Ecol.* **560**, 151820. 10.1016/j.jembe.2022.151820 (2023).

[26] Bellantuono, A. J., Granados-Cifuentes, C., Miller, D. J., Hoegh-Guldberg, O. & Rodriguez-Lanetty, M. Coral thermal tolerance: tuning gene expression to resist thermal stress. *PLoS ONE*. **7**, e50685. 10.1371/journal.pone.0050685 (2012a). 23226355 10.1371/journal.pone.0050685 PMC3511300

[27] Bay, R. A. & Palumbi, S. R. Rapid acclimation ability mediated by transcriptome changes in reef-building corals. *Genome Biol. Evol.* **7**, 1602–1612. 10.1093/gbe/evv085 (2015). 25979751 10.1093/gbe/evv085 PMC4494073

[28] Drury, C., Dilworth, J., Majerová, E., Caruso, C. & Greer, J. B. Expression plasticity regulates intraspecific variation in the acclimatization potential of a reef-building coral. *Nat. Commun.* **13**, 4790. 10.1038/s41467-022-32452-4 (2022). 35970904 10.1038/s41467-022-32452-4 PMC9378650

[29] Bellantuono, A. J., Hoegh-Guldberg, O. & Rodriguez-Lanetty, M. Resistance to thermal stress in corals without changes in symbiont composition. *Proc. Biol. Sci.* **279**, 1100–1107. 10.1098/rspb.2011.1780 (2012). 10.1098/rspb.2011.1780 PMC3267153 21976690

[30] Majerová, E. & Drury, C. Thermal preconditioning in a reef-building coral alleviates oxidative damage through a BI-1-mediated antioxidant response. *Front. Mar. Sci.* **9**10.3389/fmars.2022.971332 (2022).

[31] Gibbin, E. M. et al. Short-term thermal acclimation modifies the metabolic condition of the coral holobiont. *Front. Mar. Sci.* **5**10.3389/fmars.2018.00010 (2018).

[32] Brown, K. T., Martynek, M. P. & Barott, K. L. Local habitat heterogeneity rivals regional differences in coral thermal tolerance. *Coral Reefs*. **43**, 571–585. 10.1007/s00338-024-02484-x (2024).

[33] Helgoe, J., Davy, S. K., Weis, V. M. & Rodriguez-Lanetty, M. Triggers, cascades, and endpoints: connecting the Dots of coral bleaching mechanisms. *Biol. Rev. Camb. Philos. Soc.* **99**, 715–752. 10.1111/brv.13042 (2024). 38217089 10.1111/brv.13042

[34] Chiplunkar, S. Acclimatization of anemonia viridis (Forskäl 1775) by thermal preconditioning: potential role of catalase. *Plymouth Student Sci.* **7**, 13 (2014).

[35] Lesser, M. P. Oxidative stress in marine environments: biochemistry and physiological ecology. *Annu. Rev. Physiol.* **68**, 253–278. 10.1146/annurev.physiol.68.040104.110001 (2006). 16460273 10.1146/annurev.physiol.68.040104.110001

[36] Deponte, M. Glutathione catalysis and the reaction mechanisms of glutathione-dependent enzymes. *Biochim. Biophys. Acta*. **1830**, 3217–3266. 10.1016/j.bbagen.2012.09.018 (2013). 23036594 10.1016/j.bbagen.2012.09.018

[37] Seveso, D. et al. Investigating the heat shock protein response involved in coral bleaching across scleractinian species in the central red sea. *Coral Reefs*. **39**, 85–98. 10.1007/s00338-019-01878-6 (2020).

[38] Montalbetti, E. et al. Manganese benefits heat-stressed corals at the cellular level. *Front. Mar. Sci.* **8**, 681119. 10.3389/fmars.2021.681119 (2021).

[39] Evensen, N. R. et al. Empirically derived thermal thresholds of four coral species along the red sea using a portable and standardized experimental approach. *Coral Reefs*. **41**, 239–252. 10.1007/s00338-022-02233-y (2022).

[40] Brown, B. E. Coral bleaching: causes and consequences. *Coral Reefs*. **21**, 1–8. 10.1007/s00338-002-0211-7 (2002).

[41] Louis, Y. D., Bhagooli, R., Kenkel, C. D., Baker, A. C. & Dyall, S. D. Gene expression biomarkers of heat stress in scleractinian corals: promises and limitations. *Comp. Biochem. Physiol. Part. C: Toxicol. Pharmacol.* **191**, 63–77. 10.1016/j.cbpc.2016.08.007 (2017). 10.1016/j.cbpc.2016.08.007 27585119

[42] Seveso, D. et al. Diel modulation of Hsp70 and Hsp60 in corals living in a shallow reef. *Coral Reefs*. **37**, 801–806. 10.1007/s00338-018-1703-0 (2018).

[43] Seveso, D. et al. The susceptibility of corals to thermal stress by analyzing Hsp60 expression. *Mar. Environ. Res.* **99**, 69–75 (2014). 24999860 10.1016/j.marenvres.2014.06.008

[44] Seveso, D. et al. Hsp60 expression profiles in the reef-building coral seriatopora caliendrum subjected to heat and cold shock regimes. *Sci. Rep.* **6**, 30123. 10.1038/srep30123 (2016). 27183199 10.1016/j.marenvres.2016.05.007

[45] Mayer, M. P. & Bukau, B. Hsp70 chaperones: cellular functions and molecular mechanism. *Cell. Mol. Life Sci.* **62**, 670–684. 10.1007/s00018-004-4464-6 (2005). 15770419 10.1007/s00018-004-4464-6 PMC2773841

[46] Daugaard, M., Rohde, M. & Jäättelä, M. The heat shock protein 70 family: highly homologous proteins with overlapping and distinct functions. *FEBS Lett.* **581**, 3702–3710. 10.1016/j.febslet.2007.05.039 (2007). 17544402 10.1016/j.febslet.2007.05.039

[47] Balchin, D., Hayer-Hartl, M. & Hartl, U. F. In vivo aspects of protein folding and quality control. *Science* **353**, aac4354. 10.1126/science.aac4354 (2016). 27365453 10.1126/science.aac4354

[48] Downs, C. A. et al. Oxidative stress and seasonal coral bleaching. *Free Radic Biol. Med.* **33**, 533–543. 10.1016/s0891-5849(02)00907-3 (2002). 12160935 10.1016/s0891-5849(02)00907-3

[49] Montalbetti, E. et al. Short-term microplastic exposure triggers cellular damage through oxidative stress in the soft coral *Coelogorgia Palmosa*. *Mar. Biol. Res.* **18**, 495–508 (2022).

[50] Richier, S. Symbiosis-induced adaptation to oxidative stress. *J. Exp. Biol.* **208**, 277–285. 10.1242/jeb.01368 (2005). 15634847 10.1242/jeb.01368

[51] Weis, V. M. Cellular mechanisms of cnidarian bleaching: stress causes the collapse of symbiosis. *J. Exp. Biol.* **211**, 3059–3066. 10.1242/jeb.009597 (2008). 18805804 10.1242/jeb.009597

[52] Baker, A. C., Glynn, P. W. & Riegl, B. Climate change and coral reef bleaching: an ecological assessment of long-term impacts, recovery trends and future outlook. *Estuar. Coast Shelf Sci.* **80**, 435–471. 10.1016/j.ecss.2008.09.003 (2008).

[53] Zhang, W., Liu, R., Chen, Y., Wang, M. & Du, J. Crosstalk between oxidative stress and exosomes. *Oxid. Med. Cell. Longev.* 3553617. 10.1155/2022/3553617 (2022). 10.1155/2022/3553617 PMC9448575 36082080

[54] Krueger, T. et al. Differential coral bleaching—Contrasting the activity and response of enzymatic antioxidants in symbiotic partners under thermal stress. *Comp. Biochem. Physiol. Mol. Integr. Physiol.* **190**, 15–25. 10.1016/j.cbpa.2015.08.012 (2015). 10.1016/j.cbpa.2015.08.012 26310104

[55] Dias, M. et al. Long-term exposure to increasing temperatures on scleractinian coral fragments reveals oxidative stress. *Mar. Environ. Res.* **150**, 104758 (2019). 31301459 10.1016/j.marenvres.2019.104758

[56] Cziesielski, M. J., Schmidt-Roach, S. & Aranda, M. The past, present, and future of coral heat stress studies. *Ecol. Evol.* **9**, 10055–10066. 10.1002/ece3.5576 (2019). 31534713 10.1002/ece3.5576 PMC6745681

[57] Contardi, M. et al. Exploration of biomaterial-based solutions for coral reef restoration using biopolymer scaffolds. *ACS Appl. Mater. Interfaces*. **15**, 33916–33931 (2023). 37376819 10.1021/acsami.3c01166 PMC10360034

[58] Kültz, D. Molecular and evolutionary basis of the cellular stress response. *Annu. Rev. Physiol.* **67**, 225–257. 10.1146/annurev.physiol.67.040403.103635 (2005). 15709958 10.1146/annurev.physiol.67.040403.103635

[59] DeSalvo, M. K. et al. Differential gene expression during thermal stress and bleaching in the Caribbean coral *Montastraea faveolata*. *Mol. Ecol.* **17**, 3952–3971. 10.1111/j.1365-294X.2008.03879.x (2008). 18662230 10.1111/j.1365-294X.2008.03879.x

[60] Voolstra, C. R. et al. Effects of temperature on gene expression in embryos of the coral *Montastraea faveolata*. *BMC Genom.* **10**, 627. 10.1186/1471-2164-10-627 (2009). 10.1186/1471-2164-10-627 PMC2807443 20030803

[61] Travesso, M., Missionário, M., Cruz, S., Calado, R. & Madeira, D. Combined effect of marine heatwaves and light intensity on the cellular stress response and photophysiology of the leather coral *Sarcophyton cf. glaucum*. *Sci. Total Environ.* **861**, 160460 (2023). 36435249 10.1016/j.scitotenv.2022.160460

[62] Ayala, A., Muñoz, M. F. & Argüelles, S. Lipid peroxidation: production, metabolism, and signaling mechanisms of malondialdehyde and 4-hydroxy-2-nonenal. *Oxid. Med. Cell. Longev.* 360438 (2014). 10.1155/2014/360438 (2014). 10.1155/2014/360438 PMC4066722 24999379

[63] Majerová, E., Carey, F. C., Drury, C. & Gates, R. D. Preconditioning improves bleaching tolerance in the reef-building coral *Pocillopora acuta* through modulations in the programmed cell death pathways. *Mol. Ecol.* **30**, 3560–3574. 10.1111/mec.15988 (2021). 34008873 10.1111/mec.15988

[64] Huang, W. et al. Short-term thermal acclimation improved the thermal tolerance of three species of scleractinian corals in the South China sea. *J. Sea Res.* **199**, 102505. 10.1016/j.seares.2024.102505 (2024).

[65] Hume, B. et al. Corals from the persian/arabian Gulf as models for thermotolerant reef-builders: prevalence of clade C3 *Symbiodinium*, host fluorescence, and *ex situ* temperature tolerance. *Mar. Pollut Bull.* **72**, 313–322. 10.1016/j.marpolbul.2012.11.032 (2013). 23352079 10.1016/j.marpolbul.2012.11.032

[66] Hume, B. et al. *Symbiodinium thermophilum* sp. nov., a thermotolerant symbiotic Alga prevalent in corals of the world’s hottest sea, the persian/arabian Gulf. *Sci. Rep.* **5**, 8562. 10.1038/srep08562 (2015). 25720577 10.1038/srep08562 PMC4342558

[67] Voolstra, C. R. et al. Standardized short-term acute heat stress assays resolve historical differences in coral thermotolerance across microhabitat reef sites. *Glob Change Biol.* **26**, 4328–4343. 10.1111/gcb.15148 (2020). 10.1111/gcb.15148 32567206

[68] Jeffrey, S. W. & Humphrey, G. F. New spectrophotometric equations for determining chlorophylls a, b, c, and c2 in higher plants, algae, and natural phytoplankton. *Biochem. Physiol. Pflanzen*. **167**, 191–194 (1975).

[69] Louis, Y. D., Dyall, S. D. & Bhagooli, R. Coast-reef scale physiological responses of *Acropora muricata* harboring *Symbiodinium* clade A. *Proc. 13th Int. Coral Reef Symp.* (2016).

[70] Ladrière, O. et al. Natural Spatial variability of algal endosymbiont density in the coral *Acropora globiceps*: a small-scale approach along environmental gradients around Moorea (French Polynesia). *J. Mar. Biol. Assoc. U K*. **94**, 65–74 (2014).

[71] Isa, V. et al. Physical and cellular impact of environmentally relevant microplastic exposure on thermally challenged *Pocillopora damicornis* (Cnidaria, Scleractinia). *Sci. Total Environ.* **918**, 170651. 10.1016/j.scitotenv.2024.170651 (2024). 38320710 10.1016/j.scitotenv.2024.170651

[72] Yu, X., Huang, B., Zhou, Z., Tang, J. & Yu, Y. Involvement of caspase3 in the acute stress response to high temperature and elevated ammonium in stony coral Pocillopora damicornis. *Gene* **637**, 108–114. 10.1016/j.gene.2017.09.040 (2017). 28942037 10.1016/j.gene.2017.09.040

[73] Zhang, Y., Zhou, Z., Wang, L. & Huang, B. Transcriptome, expression, and activity analyses reveal a vital heat shock protein 70 in the stress response of stony coral *Pocillopora damicornis*. *Cell. Stress Chaperon*. **23**, 711–721. 10.1007/s12192-018-0883-4 (2018). 10.1007/s12192-018-0883-4 PMC6045544 29435724

[74] Kvitt, H., Rosenfeld, H., Zandbank, K. & Tchernov, D. Regulation of apoptotic pathways by *Stylophora pistillata* (Anthozoa, Pocilloporidae) to survive thermal stress and bleaching. *PLoS ONE*. **6**, e28665. 10.1371/journal.pone.0028665 (2011). 22194880 10.1371/journal.pone.0028665 PMC3237478

[75] Mayfield, A. B., Wang, Y. B., Chen, C. S., Lin, C. Y. & Chen, S. H. Compartment-specific transcriptomics in a reef-building coral exposed to elevated temperatures. *Mol. Ecol.* **23**, 5816–5830. 10.1111/mec.12982 (2014). 25354956 10.1111/mec.12982 PMC4265203

[76] Putnam, H. M., Mayfield, A. B., Fan, T. Y., Chen, C. S. & Gates, R. D. The physiological and molecular responses of larvae from the reef-building coral *Pocillopora damicornis* exposed to near-future increases in temperature and *p*CO2. *Mar. Biol.* **160**, 2157–2173. 10.1007/s00227-012-2129-9 (2013).

[77] Kvitt, H., Rosenfeld, H. & Tchernov, D. The regulation of thermal stress-induced apoptosis in corals reveals high similarities in gene expression and function to higher animals. *Sci. Rep.* **6**, 30359. 10.1038/srep30359 (2016). 27460544 10.1038/srep30359 PMC4961959

[78] Livak, K. J. & Schmittgen, T. D. Analysis of relative gene expression data using real-time quantitative PCR and the 2(-Delta delta C(T)) method. *Methods* **25**, 402–408. 10.1006/meth.2001.1262 (2001). 11846609 10.1006/meth.2001.1262

[79] Ferrara, E. F., Roik, A., Ziegler, M., Voolstra, C. R. & Aranda, M. *Ex situ* thermal preconditioning modulates coral physiology and enhances heat tolerance: a multispecies perspective for active restoration. *ACS Environ. Au*. **4**, 148–163. 10.1021/acsenvironau.4c00035 (2024). 10.1021/acs.est.4c08640 PMC12060272 40279456

[80] Louis, Y. D. et al. Local acclimatisation-driven differential gene and protein expression patterns of Hsp70 in *Acropora muricata*: implications for coral tolerance to bleaching. *Mol. Ecol.* **29**, 4382–4394 (2020). 32967057 10.1111/mec.15642

[81] Seneca, F.O. & Palumbi, S. R. The role of transcriptome resilience in resistance of corals to bleaching. *Mol. Ecol.* **7**, 1467–1484. 10.1111/mec.13125 (2015). 10.1111/mec.13125 25728233

[82] Barshis, D. et al. (ed, J.) Genomic basis for coral resilience to climate change. *Proc. Natl. Acad. Sci. U S A* **110** 1387–1392 (2013). 23297204 10.1073/pnas.1210224110 PMC3557039

[83] Veal, C. J., Carmi, M., Fine, M. & Hoegh-Guldberg, O. Increasing the accuracy of surface area Estimation using single wax dipping of coral fragments. *Sci. Rep.* **29**, 893–897. 10.1007/s00338-010-0647-9 (2010).

[84] Vance, P. G., Keele, B. B. & Rajagopalan, K. V. Superoxide dismutase from *Streptococcus mutans*: isolation and characterization of two forms of the enzyme. *J. Biol. Chem.* **247** (15), 4782–4786. 10.1016/S0021-9258(19)44979-X (1972). 4559499

[85] Bergmeyer, H. U., Bergmeyer, J. & Grassl, M. *Methods of enzymatic analysis* I (Fundamentals). (1983).

[86] Wang, Y., Oberley, L. W. & Murhammer, D. W. Antioxidant defense systems of two lipidopteran insect cell lines. *Free Radic Res.* **30** (11), 1254–1262. 10.1016/S0891-5849(01)00520-2 (2001). 10.1016/s0891-5849(01)00520-2 11368923

[87] Seveso, D. et al. Up-regulation of Hsp60 in response to skeleton eroding band disease but not by algal overgrowth in the scleractinian coral *Acropora muricata*. *Mar. Environ. Res.* **78**, 34–39. 10.1016/j.marenvres.2012.03.008 (2012). 22552233 10.1016/j.marenvres.2012.03.008

[88] Clarke, K. R. & Gorley, R. N. *PRIMER v7: User Manual/Tutorial* (PRIMER E, 2015).

[89] Anderson, M. J., Gorley, R. N., Clarke, K. R. & PERMANOVA + for, P. R. I. M. E. R. *Guide To Software and Statistical Methods* (PRIMER E, 2008).

