# Supplementary material for: A multi-molecular biomarker assessment of thermal preconditioning in two scleractinian coral species
Source: Sci Rep. 2025 Oct 6;15:34785. doi: 10.1038/s41598-025-18617-3 (PMC12500857; doi:10.1038/s41598-025-18617-3)
Supplement: Supplementary file 1 — Supplementary Material 1 [file 41598_2025_18617_MOESM1_ESM.docx]

**S1. Technical description of the Aquaria experimental set-up at the Genoa Aquarium (Isa et al., 2024)**

The coral colonies sampled come from the tanks of the Genoa Aquarium, where the water system collects seawater from 200 m outside the Foranea dam of the port of Genoa at 50 m depth. The collected water is pumped through the filtration system made of 2 sand filters and one UV filter, used for disinfection. After the filtration, the seawater is stored inside 4 accumulation tanks (200 m3 each). If the results of the analyses show that the chemical-physical parameters (Ph, salinity, ammonia, nitrites, nitrates, and phosphates) are optimal for the aquarium, the seawater of one accumulation tank is pumped into a mixing basin, where the water is kept in constant motion. After further UV filtration, the water is pumped from the mixing basin to all the tanks of the aquarium. During the day from 8:00 to 16:00, the water is pumped from the mixing basin to the aquarium tanks with a flow of 1 L every 30 seconds, so the tanks are considered as a semi-open system (the tanks are considered as a closed system from 16:00 to 8:00). In the tanks, used for the sampling (3 x 1 x 0.7 m; 3100 L; composed by acrylic and glass resin) in which the experiment was carried out, the water is uptaken by a pump (Astralpool, Victoria Plus) with a 24-hour flow rate of 8 m3/h (to ensure complete water change every about 30 minutes) and reinserted into the tank after passing through the filtration system. The filtration system is composed of a sand filter (Astralpool Artic, filtering particles from 0.4 to 2 mm) and a UV filter (Panaque 750 s AB 4 lamps of 40W). The water passage through the UV filter is instantaneous since water passes with a flow equal to 8 m3 per hour. Two liters of water containing a solution of the algae Tetra selmis and zooplankton belonging to the Phylum Rotifera (the average concentration of zooplankton is 250 individuals/ml and the average dimension is 0.5 mm) are placed daily inside the tanks in order to feed the corals. Both Algae and zooplankton are farmed inside 80 L cylindrical tanks made of plexiglass. Furthermore, twice a week 20 g of food mixture are daily in the tanks to feed the corals, this mixture is composed of 70% of silverside fishes (5 cm in length) and 30% carrots, while the next day the mixture is composed by 70% of mussels and 30% of courgettes. In order to facilitate the calcification of the coral skeleton 50 L of water containing 500 g of calcium hydroxide were added gradually (drop by drop) every night inside the tanks used for the sampling (3100 L tank).


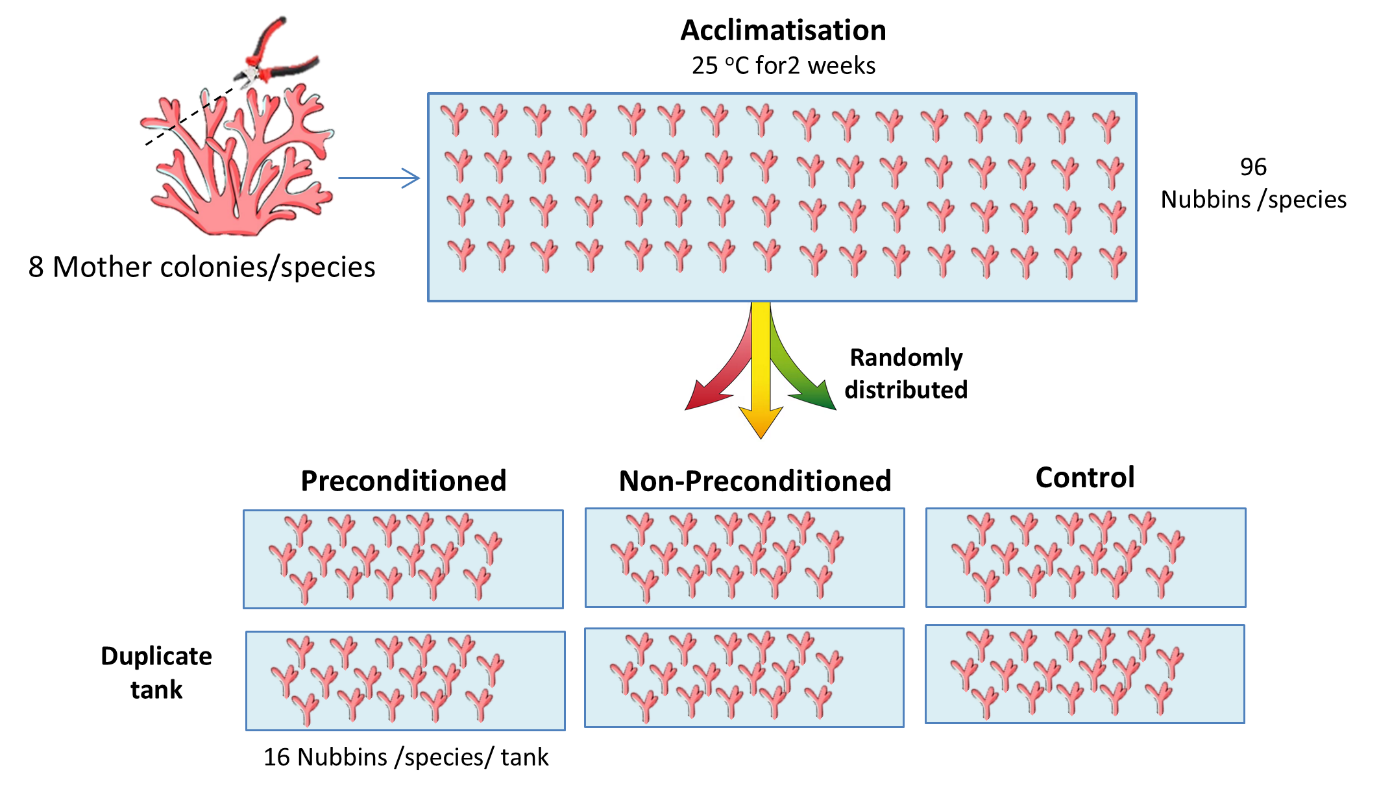


**Figure S1. Experimental design.** A total of 8 coral colonies per species were fragmented to produce 96 nubbins per species. Nubbins were acclimated together for two weeks at 25°C in a single tank. Following acclimation, nubbins were randomly assigned to three experimental treatments (each in duplicate tanks; 3 treatments × 2 replicate tanks). The schematic illustrates the design for one species; the same procedure was performed simultaneously for two species. Each tank contained *n* = 16 nubbins per species.

**S2. Chlorophyll *a* and *c2* quantification**

Coral tissue was blasted off from frozen coral fragments using filtered compressed air (Voolstra et al, 2020). Chlorophyll was extracted from tissue following Louis et al., (2016) and Isa et al., (2024). Briefly, the tissue slurry was homogenised and centrifuged at 3600 g for 4 minutes and the remaining pellet was incubated in 100% acetone for 24 h in the dark at 4 °C. Absorbance of acetone extract was measured at 630, 663, and 750 nm. Chlorophyll concentration was calculated using dinoflagellate-specific equations (Jeffrey and Humphrey, 1975) and normalized to coral surface area.

The remaining coral skeletons were immersed in 10% bleach and allowed to dry for 48 hours. The surface area of the coral fragments was determining using the the paraffin wax dipping method, following the procedure outlined by Veal et al. (2010). The weight change resulting from the wax coating was compared to a standard curve generated from dipped clay cylinders with known surface areas. This comparison enabled the calculation of the skeletal surface area for each coral fragment.

**S3. Analysis of the enzymatic activities**

3.1 *Protein extraction*

Coral fragments were ground using a pre-chilled mortar and pestle and homogenized in 750 μl lysis buffer (Tris–HCl 50 mM, pH 7.4, NaCl 150 mM, glycerol 10%, NP40 detergent 1%, EDTA 5 mM) containing 1 mM phenylmethylsulfonylfluoride (Sigma-Aldrich). Samples were then centrifuged (15 min, 14000 rpm) to allow phases separation. The supernatant was sampled and frozen immediately (- 80°C) until subsequent assays. Total protein content of each sample was determined through the Bradford method using bovine serum albumin (BSA) as a reference to design a calibration curve.

3.2 *Superoxide dismutase activity assay*

Superoxide dismutase activity (SOD) was assessed according to Vance et al. (1972). As SOD competes with ferricytochrome c for oxygen radicals, its activity was detected as the ability to inhibit the reduction of ferricytochrome c by O_2_^-^ generated from the xanthine/xanthine oxidase system. For the reaction mix, the following reagents (purchased from Sigma-Aldrich), ferricytochrome c 0.01 mM, EDTA 0.1 mM, xanthine 0.01 mM, and xanthine oxidase 0.0061 U were used in a final volume of 1 ml. Different volumes of each sample were tested and added to the reaction mix to determine the 50% inhibition of the reaction rate. The rate of reduction of ferricytochrome c was followed spectrophotometrically at 550 nm, 25°C, through a Varian Cary 50 Scan Spectrophotometer (Agilent Technologies). Under the above conditions, one unit of SOD was defined as the amount of enzyme inhibiting the reduction of ferricytochrome c by 50%. Results are expressed as units (U) of enzyme per mg of proteins.

3.3 *Catalase activity assay*

Catalase (CAT) activity was assessed by considering the peroxidatic function of the enzyme. The method is based on the degradation of hydrogen peroxide (H_2_O_2_) by the enzyme, as previously described in Bergmeyer and Grassl (1983). The reaction solution (containing 50 mM sodium phosphate buffer pH 7.5, 12 mM H_2_O_2_) was mixed in a 1 mL cuvette with different volumes of sample, and the decrease of H_2_O_2_ was followed spectrophotometrically at 240 nm (Varian Cary 50 Scan spectrophotometer, Agilent Technologies). Results are expressed as units (U) of enzyme per mg of proteins, and in this case, U refers to k, the first order kinetic constant (min^-1^), as previously described (Aebi 1984).

3.4 *Glutathione reductase activity assay*

The enzymatic assay of glutathione reductase (GR) was performed according to Wang et al. (2001). The activity of GR was evaluated through the spectrophotometric detection of the absorbance at 340 nm (Varian Cary 50 Scan spectrophotometer, Agilent Technologies) of NADPH oxidation to NADP+ reaction, which occurs in conjunction with the glutathione reduction, and is proportional to the decrease in absorbance over time. NADPH reaction was initially detected in the reaction mix (containing 0.1 M potassium phosphate buffer pH 7.6, 0.16 mM NADPH, 1 mg ml^−1^ BSA, and 4.6 mM oxidized glutathione), and then adding different volumes of sample. GR activity was obtained from the difference of the two absorbance values. One unit of GR activity was defined as the oxidation of 1 nmol NADPH/min at 25°C. Results are expressed as units (U) of enzyme per mg of proteins.

**S4. Lipid peroxidation**

Lipid peroxidation levels were measured by assessing malondialdehyde (MDA) concentrations, using a commercially available MDA assay kit (Bioxytech LPO-586, Oxis International, USA). The method is based on the reaction of a chromogenic reagent, *N* -methyl-2-phenylindole, with MDA at 45°C. Specifically, frozen coral branches (approximately 1 g each) were reduced into powder with pre-chilled mortar and pestle and homogenized in 1 ml of 20 mM phosphate buffer, pH 7.4. To prevent sample oxidation, 10 μl of 0.5 M butylated hydroxytoluene in acetonitrile were added to 1 ml of tissue homogenate. Following sample centrifugation (3000 × *g* at 4°C for 10 min), an aliquot of supernatant was used for protein determination using the Bradford method. The subsequent assay procedure (hydrochloric acid solvent procedure) was performed according to the manufacturer’s instructions. The blue product was quantified by measuring absorbance at 586 nm (Gérard-Monnier et al., 1998). Results are expressed as μmol of MDA per μg of proteins.

**S5. Analysis of the Hsp70 expression**

5.1 *Protein extract preparation and Western Blot analysis*

Frozen *P. damicornis* fragments were ground using a pre-chilled mortar and pestle and homogenised in SDS-buffer (0.0625 M Tris–HCl, pH 6.8, 10% glycerol, 2.3% SDS, 5% 2-mercaptoethanol) containing 1 mM phenylmethylsulfonylfluoride (Sigma-Aldrich), and complete EDTA-free protease inhibitors cocktail (Roche Diagnostic). Extracts were stored at -80°C until further processing. Aliquots were used to determine total protein concentrations through Bio-Rad protein assay kit (Bio-Rad Laboratories). An equal amount of proteins for each sample was separated by SDS-PAGE on 8% polyacrylamide gels (Vai et al., 1986), then run in duplicates using a Mini-Protean Tetra Cell (Bio-Rad Laboratories). After the electrophoresis, the first gel was stained with Coomassie Brilliant Blue to visualize total proteins, while the second gel was electroblotted onto nitrocellulose membrane (Amersham Protran 0.45 mm) for Western Blot analysis as previously described (Seveso et al., 2012). Correct protein transfer was confirmed by Ponceau S Red (Sigma-Aldrich) staining of filters. Following saturation with TBS and 5% skimmed milk, filters were probed with the following primary antibodies: anti-Hsp70 monoclonal antibody (IgG2a mouse clone BB70, SPA-822, Enzo Life Sciences) diluted 1:1000 in TBS-0.1% Tween 20 and 5% skimmed milk, and anti-β-Actin monoclonal antibody (IgG1k mouse clone C4, MAB1501, Millipore) diluted 1:3000. After being washed three times with fresh TBS-0.1% Tween 20 (15 min each), filters were incubated with anti-mouse IgG polyclonal secondary antibodies conjugated with horseradish peroxidase (ADI-SAB-100, Enzo Life Sciences) diluted 1:1000 for Hsp70 and 1:15000 for β–Actin in TBS-0.1% Tween 20 and 5% skimmed milk. Western blots were developed using Pierce ECL Western Blotting Substrate followed by exposition of filters to Amersham Hyperfilm ECL.

5.2 *Densitometric Analysis*

Densitometric analyses were performed as described by Seveso et al., (2013). Films were scanned on a Bio-Rad GS-800 calibrated imaging densitometer and the pixel density of the scanned bands were quantified with the ImageJ free software of the NIH Image software package (National Institutes of Health, Bethesda, Md.). The scanned intensity of the bands of Hsp70 was normalized against the intensity of the β-Actin ones, which was used as an internal loading control since in all our experiments the β-Actin level did not display significant changes. The densitometric data were expressed as relative levels (arbitrary units).

**S6. Gene expression analysis**

Total RNA was extracted as described in Isa et al., (2024) Briefly, the coral tissue was blasted off 0.3-0.5 g of coral fragments in a pre-cooled mortar, using filtered compressed air for a maximum of 3 minutes (Voolstra et al., 2020). The tissue was immediately ground under liquid nitrogen and 600 µl of lysis buffer (RLT Buffer containing β-mercaptoethanol) was added. The tissue lysate was homogenised by passing it through a 20-gauge (0.9 mm) sterile plastic syringe. RNA extractions were then continued using the Qiagen RNA Mini kit (Qiagen) following the manufacturer's instructions for purification of total RNA from animal tissues. DNA contamination was removed using the DNase I Set (Zymo Research) in combination with the RNA Clean & Concentrator-25 kit (Zymo Research) according to the manufacturer's protocol. RNA quality was checked by examining with gel electrophoresis for clear, sharp bands of ribosomal RNAs. RNA concentration was estimated using Qubit (RNA Broad Range Assay Kit, Thermo Fisher Scientific).

Real-Time qPCR was performed using the QuantiNova SYBR Green RT-PCR Kit (Qiagen) according to the manufacturer's instructions. Reactions were performed, in triplicate, using a CFX Connect Real-Time PCR System (Biorad) using the following thermal cycle: reverse transcription 10 min at 50 °C, PCR initial heat activation 2 min, 40 cycles of 95°C for 5 s (denaturation) then 60°C for 10 s (combined annealing/extension). At the end of the cycle, a melt curve was performed from 60°C to 95°C to ensure a single peak was observed indicating absence of non-specific PCR products.

The *ef* gene has been widely employed as an internal control in numerous studies due to its consistent expression levels across various experimental stress conditions, including heat stress (Yu et al., 2017; Zhang et al., 2018; Isa et al., 2024). Similarly, *18S rRNA* has been validated as a reliable internal control under similar conditions (Kvitt et al., 2011).

The sequences of the PCR primers used are given in the main text, Table 1. Primer efficiency was assessed through a serial dilution (ranging from 1 in 10) of a mixed RNA sample, with efficiencies consistently falling between 0.94 and 1.10. Relative changes in gene expression were quantified using the 2−ΔΔCt method (Livak & Schmittgen, 2001). All calculations were performed in R Studio, and gene expression results are presented as log2 fold-changes relative to the control group prior to the onset of acute stress.

**Table S1.** Results of the two-factor univariate PERMANOVA testing the effects of the different treatments (C, PC and NPC) and sampling time points (Before stress, Day 1, Day 3, Day 10) on the levels of the different biomarkers analysed in *Pocillopora damicornis,* and obtained by permutations (perm) for each group. Significant *p*-values (*p* < 0.05) are shown in bold.

| **Source of variation** | ***df*** | **SS** | **MS** | **Pseudo *F*** | **p(MC)** |
| --- | --- | --- | --- | --- | --- |
| ***Chl a*** |  |  |  |  |  |
| Treatment | 2 | 0.32029 | 0.16014 | 5.0765 | **0.008** |
| Time | 3 | 0.16673 | 0.055576 | 1.7617 | 0.1693 |
| Treatment x Time | 6 | 0.2509 | 0.041817 | 1.3256 | 0.2625 |
| Residual | 57 | 1.7981 | 0.031546 |  |  |
| Total | 68 | 2.5367 |  |  |  |
| ***Chl c2*** |  |  |  |  |  |
| Treatment | 2 | 0.9621 | 0.48105 | 27.009 | **0.0001** |
| Time | 3 | 0.5445 | 0.1815 | 10.191 | **0.0001** |
| Treatment x Time | 6 | 0.38697 | 0.06449 | 3.6211 | **0.0049** |
| Residual | 51 | 0.90835 | 0.017811 |  |  |
| Total | 62 | 2.911 |  |  |  |
| ***Symbiodinium density*** |  |  |  |  |  |
| Treatment | 2 | 0.79159 | 0.39579 | 8.8073 | **0.0002** |
| Time | 3 | 0.24309 | 0.08103 | 1.8031 | 0.1679 |
| Treatment x Time | 6 | 0.89244 | 0.14874 | 3.3098 | **0.0103** |
| Residual | 36 | 1.6178 | 0.044939 |  |  |
| Total | 47 | 3.5449 |  |  |  |
| ***Hsp70 protein*** |  |  |  |  |  |
| Treatment | 2 | 2.0798 | 1.0399 | 12.943 | **0.0002** |
| Time | 3 | 3.0814 | 1.0271 | 12.784 | **0.0001** |
| Treatment x Time | 6 | 2.2124 | 0.36874 | 4.5894 | **0.0022** |
| Residual | 36 | 2.8924 | 0.080346 |  |  |
| Total | 47 | 10.266 |  |  |  |
| ***GR*** |  |  |  |  |  |
| Treatment | 2 | 0.38928 | 0.19464 | 12.509 | **0.001** |
| Time | 3 | 0.18182 | 0.060607 | 3.895 | **0.0511** |
| Treatment x Time | 6 | 0.1827 | 0.03045 | 1.9569 | 0.1689 |
| Residual | 84 | 1.30711 | 0.01556 |  |  |
| Total | 95 | 2.0609 |  |  |  |
| ***CAT*** |  |  |  |  |  |
| Treatment | 2 | 0.33033 | 0.16516 | 11.279 | **0.0002** |
| Time | 3 | 0.477841 | 0.15928 | 10.877 | **0.0001** |
| Treatment x Time | 6 | 0.585426 | 0.097571 | 6.6628 | **0.0003** |
| Residual | 54 | 0.790783 | 0.014644 |  |  |
| Total | 65 | 2.2037 |  |  |  |
| ***SOD*** |  |  |  |  |  |
| Treatment | 2 | 0.56996 | 0.28498 | 25.346 | **0.0001** |
| Time | 3 | 0.087565 | 0.02918 | 2.596 | 0.0638 |
| Treatment x Time | 6 | 0.87243 | 0.1454 | 12.932 | **0.0001** |
| Residual | 51 | 0.57341 | 0.011243 |  |  |
| Total | 62 | 2.0301 |  |  |  |
| ***LPO*** |  |  |  |  |  |
| Treatment | 2 | 0.75308 | 0.37654 | 12.014 | **0.0002** |
| Time | 3 | 4.0973 | 1.3658 | 43.575 | **0.0001** |
| Treatment x Time | 6 | 2.2628 | 0.37713 | 12.032 | **0.0001** |
| Residual | 35 | 1.097 | 0.031343 |  |  |
| Total | 46 | 8.2816 |  |  |  |
| ***hsp70 gene*** |  |  |  |  |  |
| Treatment | 2 | 0.3538 | 0.1769 | 42.987 | **0.0001** |
| Time | 3 | 0.22758 | 0.07586 | 18.434 | **0.0001** |
| Treatment x Time | 6 | 0.38926 | 0.06487 | 15.765 | **0.0001** |
| Residual | 53 | 0.21811 | 0.004115 |  |  |
| Total | 64 | 1.2125 |  |  |  |

**Table S2.** Results of PERMANOVA pair-wise comparisons for all biomarker analysed in *P. damicornis* between sampling time points in each treatment (A) and between treatments for each sampling time point (B) obtained by permutations (perm) for each group. Significant p-values (*p* < 0.05) are in shown bold.

**Table S3.** Results of the two-factor univariate PERMANOVA testing the effects of the different treatments (C, PC and NPC) and sampling time points (Time; Before stress, Day 1, Day 3, Day 10) on the levels of the different biomarkers analysed in *Stylophora pistillata,* obtained by permutations (perm) for each group. Significant *p*-values (*p* < 0.05) are shown in bold

| **Source of variation** | ***df*** | **SS** | **MS** | **Pseudo *F*** | **p(MC)** |
| --- | --- | --- | --- | --- | --- |
| ***Chla*** |  |  |  |  |  |
| Treatment | 2 | 3.5664 | 1.7832 | 29.507 | **0.0001** |
| Time | 3 | 4.6836 | 1.5612 | 25.833 | **0.0001** |
| Treatment x Time | 6 | 2.4915 | 0.41524 | 6.871 | **0.0001** |
| Residual | 84 | 5.0764 | 0.060434 |  |  |
| Total | 95 | 15.818 |  |  |  |
| ***Chl2*** |  |  |  |  |  |
| Treatment | 2 | 0.33903 | 0.16952 | 4.517 | **0.0169** |
| Time | 3 | 0.88992 | 0.29664 | 7.9045 | **0.0005** |
| Treatment x Time | 6 | 0.58643 | 0.097738 | 2.6044 | **0.0269** |
| Residual | 84 | 3.1524 | 0.037528 |  |  |
| Total | 95 | 4.9678 |  |  |  |
| ***Symbiodinium density*** |  |  |  |  |  |
| Treatment | 2 | 0.057561 | 0.028781 | 9.1751 | **0.0007** |
| Time | 3 | 0.11797 | 0.039322 | 12.536 | **0.0001** |
| Treatment x Time | 6 | 0.09484 | 0.015808 | 5.0395 | **0.0007** |
| Residual | 50 | 0.15684 | 0.003136 |  |  |
| Total | 61 | 0.43367 |  |  |  |
| ***Hsp70 protein*** |  |  |  |  |  |
| Treatment | 2 | 0.70943 | 0.35471 | 11.954 | **0.0001** |
| Time | 3 | 0.90637 | 0.30121 | 10.182 | **0.0001** |
| Treatment x Time | 6 | 0.36854 | 0.061423 | 2.07 | 0.0819 |
| Residual | 36 | 1.0682 | 0.029673 |  |  |
| Total | 47 | 3.05266 |  |  |  |
| ***GR*** |  |  |  |  |  |
| Treatment | 2 | 0.38928 | 0.19464 | 12.509 | **0.001** |
| Time | 3 | 0.18182 | 0.060607 | 3.895 | 0.0511 |
| Treatment x Time | 6 | 0.1827 | 0.03045 | 1.9569 | 0.1689 |
| Residual | 84 | 1.30711 | 0.01556 |  |  |
| Total | 95 | 2.0609 |  |  |  |
| ***CAT*** |  |  |  |  |  |
| Treatment | 2 | 1.127 | 0.5635 | 9.9326 | **0.0003** |
| Time | 3 | 1.29281 | 0.43092 | 7.5956 | **0.0005** |
| Treatment x Time | 6 | 1.6724 | 0.27874 | 4.9132 | **0.0003** |
| Residual | 78 | 4.4251 | 0.056732 |  |  |
| Total | 89 | 8.569 |  |  |  |
| ***SOD*** |  |  |  |  |  |
| Treatment | 2 | 0.23265 | 0.11632 | 11.067 | **0.0002** |
| Time | 3 | 0.29725 | 0.099084 | 9.4266 | **0.0001** |
| Treatment x Time | 6 | 0.33303 | 0.055506 | 5.2807 | **0.0001** |
| Residual | 84 | 0.88293 | 0.010511 |  |  |
| Total | 95 | 1.7459 |  |  |  |
| ***LPO*** |  |  |  |  |  |
| Treatment | 2 | 0.14034 | 0.07017 | 8.5425 | **0.0005** |
| Time | 3 | 0.028202 | 0.00940 | 1.1444 | 0.3373 |
| Treatment x Time | 6 | 0.073015 | 0.012169 | 1.4815 | 0.1937 |
| Residual | 78 | 0.64071 | 0.008214 |  |  |
| Total | 89 | 0.87548 |  |  |  |
| ***hsp70 gene*** |  |  |  |  |  |
| Treatment | 2 | 1.3847 | 0.69234 | 59.444 | **0.0001** |
| Time | 3 | 2.3249 | 0.77498 | 66.539 | **0.0001** |
| Treatment x Time | 6 | 0.81185 | 0.13531 | 11.617 | **0.0001** |
| Residual | 49 | 0.5707 | 0.011647 |  |  |
| Total | 60 | 5.4184 |  |  |  |

**Table S4.** Results of PERMANOVA pair-wise comparisons for all biomarker analysed in *P. damicornis* between sampling time points in each treatment (A) and between treatments in each sampling time point (B) obtained by permutations (perm) for each group. Significant *p*-values (*p* < 0.05) are shown in bold.
